# Inhibition of the mitochondrial citrate carrier, Slc25a1, reverts steatosis, glucose intolerance, and inflammation in preclinical models of NAFLD/NASH

**DOI:** 10.1038/s41418-020-0491-6

**Published:** 2020-01-20

**Authors:** Mingjun Tan, Rami Mosaoa, Garrett T. Graham, Anna Kasprzyk-Pawelec, Shreyas Gadre, Erika Parasido, Olga Catalina-Rodriguez, Patricia Foley, Giuseppe Giaccone, Amrita Cheema, Bhaskar Kallakury, Chris Albanese, Chunling Yi, Maria Laura Avantaggiati

**Affiliations:** 1 Georgetown University Medical Center, Lombardi Comprehensive Cancer Center, Washington, D.C. 20057 USA; 2 Department of Biochemistry, Faculty of Science, King Abdulaziz University, Jeddah, Saudi Arabia

**Keywords:** Fatty acids, Metabolic disorders

## Abstract

Nonalcoholic fatty liver disease (NAFLD) and its evolution to inflammatory steatohepatitis (NASH) are the most common causes of chronic liver damage and transplantation that are reaching epidemic proportions due to the upraising incidence of metabolic syndrome, obesity, and diabetes. Currently, there is no approved treatment for NASH. The mitochondrial citrate carrier, Slc25a1, has been proposed to play an important role in lipid metabolism, suggesting a potential role for this protein in the pathogenesis of this disease. Here, we show that Slc25a1 inhibition with a specific inhibitor compound, CTPI-2, halts salient alterations of NASH reverting steatosis, preventing the evolution to steatohepatitis, reducing inflammatory macrophage infiltration in the liver and adipose tissue, while starkly mitigating obesity induced by a high-fat diet. These effects are differentially recapitulated by a global ablation of one copy of the *Slc25a1* gene or by a liver-targeted *Slc25a1* knockout, which unravel dose-dependent and tissue-specific functions of this protein. Mechanistically, through citrate-dependent activities, Slc25a1 inhibition rewires the lipogenic program, blunts signaling from peroxisome proliferator-activated receptor gamma, a key regulator of glucose and lipid metabolism, and inhibits the expression of gluconeogenic genes. The combination of these activities leads not only to inhibition of lipid anabolic processes, but also to a normalization of hyperglycemia and glucose intolerance as well. In summary, our data show for the first time that Slc25a1 serves as an important player in the pathogenesis of fatty liver disease and thus, provides a potentially exploitable and novel therapeutic target.

## Introduction

Nonalcoholic fatty liver disease (NAFLD) and inflammatory steatohepatitis (NASH) comprise a spectrum of manifestations that span from reversible hepatic steatosis to progressive necroinflammatory and fibrotic disease that are associated with a high rate of mortality [1–4]. With the development of effective therapeutics for hepatitis C, NAFDL/NASH is predicted to become the most common cause of chronic liver disease and transplantation with a worldwide prevalence as high as 25%. Although the exact molecular mechanisms that drive the progression of NASH are still debated, there is a clear association with obesity, diabetes, and metabolic syndrome.

Due to the complexity of NASH evolution, the search for effective agents is a very active area of interest [4]. Early fatty liver disease can be corrected with dietetic adjustments, while at later stages several agents have been proposed as effective. Given the role of altered lipid metabolism and the deleterious effects of lipotoxicity, inhibitors of the lipid synthetic pathway, including inhibitors of fatty acid synthase (FASN) or acetyl-coenzyme-A carboxylase, ACC1/2, are being explored as potential therapeutics. However, paradoxically, the liver specific deletion of the *FASN* gene exacerbates steatosis, while ACC1/2 inhibition reduces steatosis, but induces hypertriglyceridemia [5, 6]. Additional NASH therapeutics comprise farnesoid receptor agonists (FXR) that promote bile acid biosynthetic pathways, the prototypes of which are obeticholic acid and GS-9674. Beneficial effects of FXR agonists include reduction of fibrosis potentially counterbalanced by the toxic effect of bile acid accumulation that causes hepatocyte cell death and cholestasis [3, 4]. Thus, the development of NASH therapeutics represents an unmet clinical need, to the point that the Food and Drug Administration has recently emphasized the necessity to identify new therapies that slow or reverse the progression of NAFLD/NASH.

The mitochondrial citrate carrier, Slc25a1, (or CIC) belongs to a family of ion transporters whose activity has been linked to several pathologic conditions including cancer, aging, and developmental disorders [7]. The human *Slc25a1* gene maps to chromosome 22.q11.2 and microdeletions of this region give raise to a group of survivable disorders known as Velo-cardio-facial and DiGeorge syndromes [8]. Various *Slc25a1* mutations, spanning throughout the coding region, have also been reported in combined D2-/L2-hydroxyglutaric aciduria, characterized by the pathological accumulation of these aberrant metabolites [9–13]. Moreover, mutations of a member of the citrate transporter family in fruit fly, *INDY*, promote longevity, suggesting that the citrate transport pathway controls life-span [14]. Finally, we and others have shown that Slc25a1 plays an important role in the metabolic rewiring of tumor cells, in chemotherapy resistance and as a mediator of inflammatory signals [15–19].

The known activity of Slc25a1 consists of the regulation of the cytoplasmic and mitochondrial pools of citrate. In the cytoplasm citrate is the precursor for lipogenesis and is also an allosteric regulator that binds to several enzymes modulating their function. Although citrate-binding motifs have not been identified yet, several proteins in the glycolytic and lipogenic pathway respond to allosteric regulation by citrate. These include the glycolytic enzyme, phosphofructokinase (PFK), which is inhibited by citrate binding, and Fructose 1,6-bisphosphatase (Fbp1) and acetyl-CoA carboxylase alpha (ACACA) that are activated by citrate [20]. In spite of these fundamental functions, a definite role of Slc25a1 in the metabolism in vivo has never been investigated.

Several Slc25a1 inhibitors have been developed throughout the years. A first-generation inhibitor, the false noncleavable citrate analog, benzene-tricarboxylate, has the potential to influence other citrate-binding proteins. A second-generation inhibitor (CTPI-1) shows no structural similarity with citrate and competitively binds to the Slc25a1 citrate-binding pocket [21], but requires high dosage for activity in vivo [16]. A third-generation inhibitor, CTPI-2, was discovered by our laboratory and provides a major improvement in the biological activity of this category of drugs, enhancing the binding activity towards Slc25a1 more than 20-fold compared with CTPI-1 and inhibiting citrate transport and tumor proliferation at doses tenfold lower [16]. The specificity of CTPI-2 for Slc25a1 was demonstrated by the lack of activity in cells harboring a knockdown of the gene. Moreover, comparative transcriptional and metabolomic profiling of cells treated with CTPI-2, harboring the Slc25a1-shRNA, or overexpressing the protein, demonstrate a stringent pattern of overlap of the affected genes/metabolites [16], implying that Slc25a1 is a *bona fide* target of CTPI-2.

In this work we have used a combination of genetic and drug-targeting approaches to ask the question of whether Slc25a1 influences NASH evolution. The results establish that Slc25a1 acts as a nodal protein and a “druggable” target in this disease.

## Materials and methods

### Extended “Materials and methods” are provided in the Supplementary Data File

#### Cells, reagents, antibodies, primers

The CTPI-2 was purchased from Enamine Ltd. The anti-Slc25a1 antibody used in immunoblot was either from Santa Cruz Biotech, (# sc-86392) employed at 1:1000 dilution or from Proteintech (15235–1-AP). Additional antibodies and their source are described in the Supplementary “Materials and methods”.

#### Mice and diets

Diet-induced obesity (DIO) C57BL/6J mice were purchased from Jackson laboratory between 4 and 6 weeks of age and were randomized to a standard laboratory chow diet (Labdiet #5053) or the high-fat diet (HFD) (Research diets D12492) with 60% calories derived from fat (lard), and 20% from sucrose.

#### CTPI-2 treatment

CTPI-2 was administered at 50 mg/kg via intraperitoneal injection at alternate days. CTPI-2 was diluted either in DMSO (at 0.2% final concentration) using DMSO as vehicle control, or in 0.47% Sodium Bicarbonate (NaHCO_3_) at a final concentration of 14 mM. 0.47% NaHCO_3_ served as vehicle control.

#### Slc25a1 strains

The original *Slc25a1* tm1a strain was purchased from Mutant Mouse Resource Research Center (MMRRC) (C57BL/6N-*Slc25a1*^*tm1a(EUCOMM)Wtsi*^, RRID:MMRRC_042258-UCD). The *Tg(Alb-cre)21Mgn* (*Alb-Cre*) mice were purchased from Jackson laboratory (#003574). *Slc25a1*^*+/−*^, *Slc25a1*^*fl/fl*^, and *Alb/Cre* mice were genotyped by conventional PCR with genomic DNA extracted from the mouse tail biopsies and agarose gel electrophoresis was used for analysis of PCR products. The PCR primers used for genotyping are indicated in Table S1.

#### RNA isolation, mRNA sequencing, and analysis

Total RNA was isolated from cell pellets using the Direct-zol RNA MiniPrep kit (Zymo Research, USA). The RNA quality and quantity were estimated by UV–Vis spectrophotometry using the NanoDrop ND-1000 spectrophotometer. RNA integrity was assessed using the Agilent RNA 6000 Nano Kit and final RNA yield and concentration was measured and normalized. RNA sequencing (RNAseq) was performed by the UCLA center for Neurobehavioral Genetics.

#### Immunohistochemistry, immunofluorescence, and NASH tissue microarrays

The tissue arrays of human NASH were obtained from XenoTech (1910017). The immunohistochemical (IHC) was performed using standard protocols on formalin-fixed sections. The Slc25a1 antibody (ProteinTech, Cat. 15235–1-AP) was used at 1/150 dilution. Consecutive sections with the primary antibody omitted were used as negative controls. For immunofluorescence, stained slides were scanned using the Vectra3 Multi-Spectral Imaging Microscope with Vectra and Phenochart software (PerkinElmer). The entire slide was scanned, and then ten regions of interest were selected at random throughout the tissue. The scanned images were analyzed with inForm software version 2.4.1.

#### Statistics

Statistical significance was assessed using both paired or unpaired, two-tailed Student *t* test. Significant differences are indicated using the standard Michelin Guide scale (*p < 0.05, significant; **p < 0.01, highly significant; ***p < 0.001, extremely significant).

## Results

### Slc25a1 expression is high in NASH livers and its inhibition reduces obesity and hepatomegalia

**Fig. 1:**
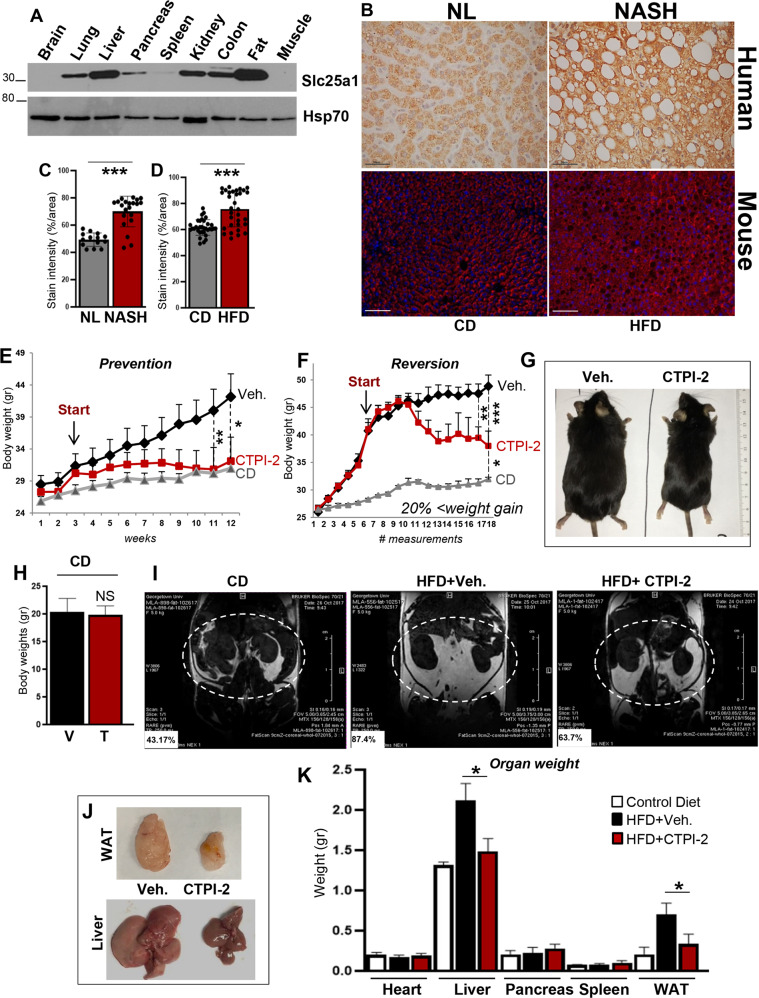
Slc25a1 levels are high in human NASH and inhibition of its activity reverts the phenotypes of mice fed a HFD. **a** Expression levels of Slc25a1 protein in the indicated organs derived from a 4-month-old male mouse. **b** Top panels: tissue microarrays derived from human normal liver (NL) and NASH patients. Bottom panels: Slc25a1 and DAPI staining in the livers of mice fed with the control (CD) or high-fat diet (HFD). **c, d** Quantification of Slc25a1 staining in human NASH livers (**c**) or murine livers (**d**) as shown in (**b**). Quantification was performed with the ImageJ program on nine NASH livers relative to three normal livers on multiple fields. **e** Body weight measurements in the prevention study. Mice were fed the HFD for 3 weeks and CTPI-2 treatment was started (indicated by arrow). **f** Body weight measurements in the reversion study. Mice fed the CD or HFD for 3 months were then randomized to receive CTPI-2 or vehicle for an additional 3 months (*n* = 3–4). Measurements were taken at regular intervals. The arrow indicates the time at which CTPI-2 treatment was started, after 12 weeks of HFD-feeding. In both cases CTPI-2 was administered at 50 mg/kg on alternate days via the intraperitoneal route. **g** Representative images of mice fed the HFD in the reversion study in the indicated treatment conditions. **h** Body weight measurements of mice fed control diet and treated with CTPI-2 for 5 months (*n* = 7). **i** MRI images of the visceral fat (circled in white) of the indicated mice. Insets numbers (in white) indicate the percentage of visceral fat calculated as threshold fat pixels versus threshold whole-body pixels in the abdominal region from every slice of the MRI image dataset (see “Materials and methods”). **j** Representative images of the hemi-lateral visceral fat (WAT) and the liver of mice fed the HFD, in the indicated treatment conditions. **k** Measurement (in grams) of the indicated organs in CD, HFD+Veh., or HFD+CTPI-2 (*n* = 3). *p ≤ 0.05, **p ≤ 0.01, ***p ≤ 0.001.

We first studied the abundance of Slc25a1 protein in murine tissues. Immunoblot experiments performed on normal mice revealed that Slc25a1 is primarily expressed in the white visceral abdominal fat (WAT) and in the liver, suggesting a role for this protein in central metabolism and energy storage (Fig. 1a). To then assess whether Slc25a1 is relevant to NAFLD/NASH, we performed IHC analysis of human tissue microarrays derived from the liver of patients with NASH, which showed higher expression levels relative to normal livers (Fig. 1b, top panels; quantified in Fig. 1c and Supplementary Fig. S1). Similarly, in mice fed with an HFD enriched in lard and sucrose, Slc25a1 expression was increased in the liver relative to control mice (Fig. 1b, bottom panels; quantified in Fig. 1d and Supplementary Fig. S2). Further, Slc25a1 was identified among genes associated with NAFLD in the comparative toxicogenomic database (https://amp.pharm.mssm.edu/Harmonizome/gene_set/+Fatty+Liver+Disease/CTD+Gene-Disease+Associations). In all, these results suggested a role for Slc25a1 in fatty liver disease.

Next, we used the well-established model of DIO of C57BL/6J mice fed with the HFD. We performed these experiments in two different settings. In the first case, the Slc25a1 inhibitor, CTPI-2, was administered approximately at the same time of initiation of the diet (prevention study, Fig. 1e). Alternatively, given that the majority of patients seeks medical attention when obesity and steatosis have already occurred, C57BL/6J mice were first fed the HFD for 3 months, and then were randomized to receive vehicle or CTPI-2 for additional 12 weeks (reversion study, Fig. 1f). To eliminate potential differences in feeding behavior, animals were kept at one mouse per cage and the food consumption was measured weekly. CTPI-2 did not prevent feeding and did not induce a starvation state (Supplementary Fig. S2A). As expected, HFD-fed mice exhibited a dramatic increase in body weight over time and treatment with CTPI-2 completely averted weight gain in the prevention study and led to significant weight loss in the reversion study (Fig. 1e–g). However, CTPI-2 did not modify body weights in mice fed with a normal diet (Fig. 1h). Magnetic resonance imaging (MRI) performed in animals enrolled in the reversion study, as well as visual examination of their abdomen confirmed that CTPI-2 particularly reduced the WAT (Fig. 1i, j). Further, upon visual examination, the liver of HFD-fed mice was pale and enlarged and CTPI-2 reverted this phenotype (Fig. 1j). Measurement of the weight of all organs confirmed that CTPI-2 selectively reduced the mass of the liver and WAT, in agreement with prevalent activities of this protein in these organs (Fig. 1k).

### CTPI-2 prevents steatohepatitis and normalizes glucose tolerance

**Fig. 2:**
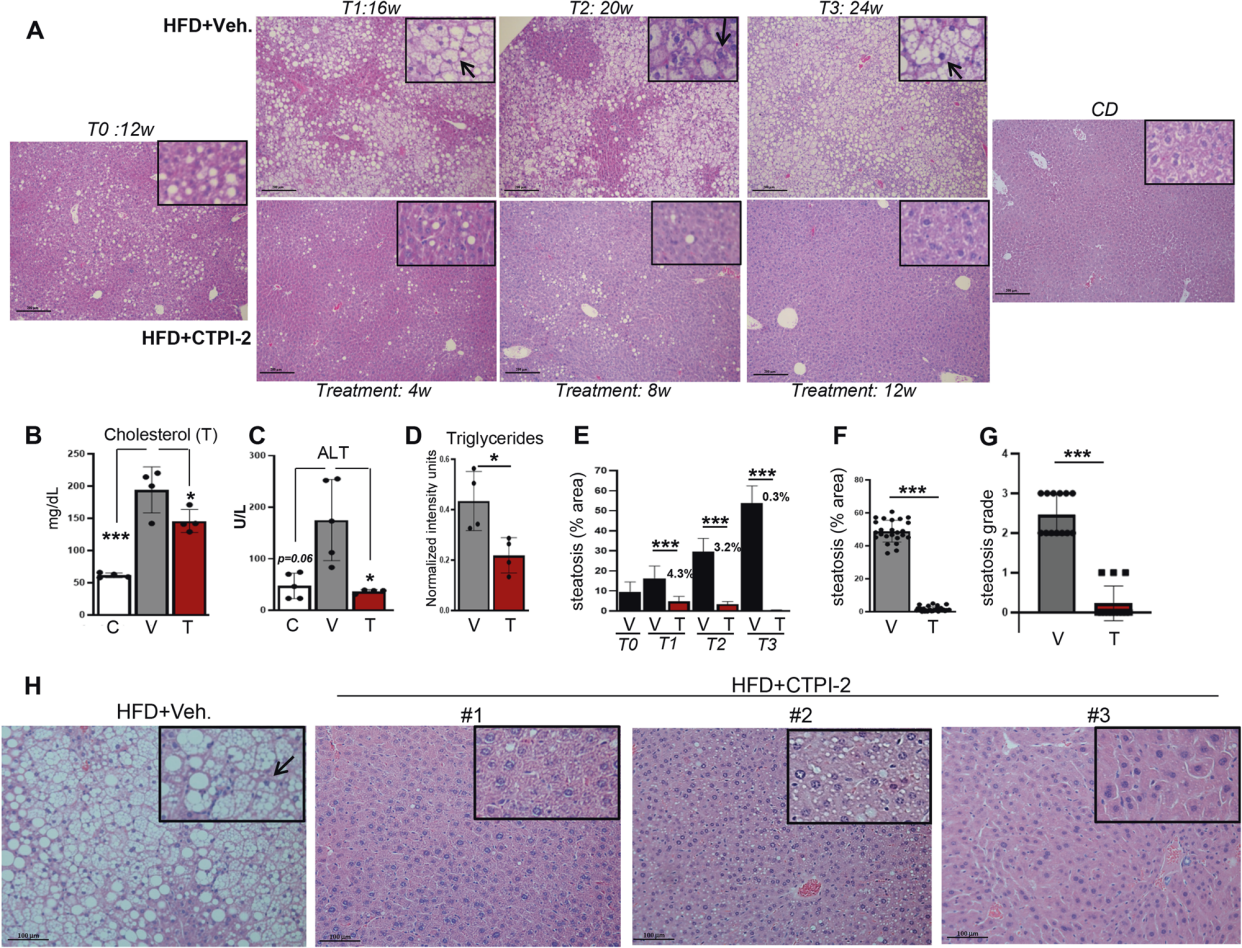
Slc25a1 inhibition with CTPI-2 ameliorates steatosis and liver injury. **a** Time-course experiments showing the livers of HFD-fed mice treated with vehicle (top panels) or with CTPI-2 (bottom panels). The weeks of diet exposure are indicated at the top; the times of treatment with CTPI-2 are at the bottom. Time 0 shows the liver histology when CTPI-2 treatment was initiated, after 12 weeks of HFD. The last panel on the right shows a normal liver derived from CD-fed mice. Rectangles show enlarged fields and arrows point to ballooning hepatocytes, when detected. **b, c** Total serum cholesterol levels and ALT levels measured with the Heska Element DC blood chemistry analyzer. **d** Serum levels of triglycerides measured with LC-MS. **e** Quantification of steatosis from the time-course experiments. The percentage of steatosis in CTPI-2 treated mice is shown on the top of each bar graph. **f, g** Quantification of liver steatosis indicated as % per field (**f**) or steatosis grade (**g**). Quantification was performed on 3–5 mice per group and on at least 2–3 fields per mouse. Steatosis grade was assessed as: grade 0: <5%; grade 1: 5–33%; grade 2: 33–50%; and grade 3: >50%. **h** Representative images of HFD-fed mice (left panel) and of three different mice treated with CTPI-2. Rectangles show enlarged fields of the images and arrows indicate hepatocyte ballooning, when present. *p ≤ 0.05, **p ≤ 0.01, ***p ≤0 0.001.

We next studied the effects of CTPI-2 in the liver of HFD-fed mice. To explore CTPI-2 activity in the most relevant therapeutic reversion setting and to determine at what stage of disease evolution CTPI-2 can be effective, we performed time course experiments over a long period of time. Hence, mice were first initiated with a HFD regimen for 3 months before CTPI-2 administration and were then sacrificed after 4, 8, and 12 weeks of treatment, corresponding to 16, 20, or 24 weeks of HFD regimen. In mice fed with the HFD the liver was filled with fatty deposits that accumulated over time in the form of micro- and macrovesicular steatosis, and the hepatocytes exhibited typical ballooning degeneration, indicating the presence of steatohepatitis (Fig. 2a). Accordingly, these mice displayed hypercholesterolemia and hepatic injury as demonstrated by the elevated levels of Alanine Aminotransferase (ALT) and CTPI-2 normalized cholesterol, ALT and triglyceride levels (Fig. 2b–d). After 3 months of exposure to the HFD, at which time CTPI-2 treatment started, steatosis was already evident and progressed over time. CTPI-2 reverted these early stages of steatosis and also prevented the evolution to steatohepatitis, identified based upon the dramatic increase of lipid accumulation, the presence of hepatocyte ballooning (Fig. 2a), the elevated levels of ALT (Fig. 2c) and the presence of perivascular inflammatory infiltration (not shown). Although we observed some extent of heterogeneity in the response of individual mice to CTPI-2 (Fig. 2h), after 3 months of CTPI-2 administration and 6 months on HFD, the livers of most of CTPI-2 treated mice appeared almost indistinguishable from that of animals fed a normal diet. In addition, when administered at the same time of initiation of the HFD, CTPI-2 nearly completely prevented steatosis, again pointing to the potential application of this drug in both the prevention and treatment settings (Supplementary Fig. S2B).

**Fig. 3:**
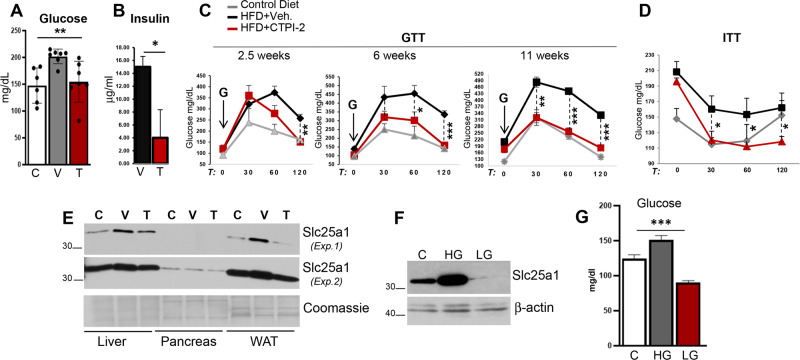
CTPI-2 normalizes glucose tolerance and insulin sensitivity. **a** Fasting glucose levels in control diet (C, white bars), HFD+Vehicle (V, grey bars), or HFD+CTPI-2 (T, red bars) mice (*n* = 3–6). **b** Insulin levels (in μg/ml) of animals treated with CTPI-2 for approximately 11 weeks (*n* = 2–3). **c** Glucose tolerance test performed at the indicated time points of CTPI-2 treatment (*n* = 3–4). Grey lines indicate control diet; black lines indicate HFD+vehicle; and red lines indicate HFD+CTPI-2. **d** Insulin tolerance test in the indicated mice groups. **e** Expression levels of Slc25a1 (shown at two different exposure times, Exp.1 and Exp.2) in the indicated organs and treatment conditions. **f** Expression levels of Slc25a1 in the visceral adipose tissue of mice fed with chow (C), high-glucose (HG), or low-glucose (LG) diets (see “Materials and methods” for diet composition). **g** Fasting glucose levels in mice fed as indicated (*n* = 4–6). *p ≤ 0.05, **p ≤ 0.01, ***p ≤ 0.001.

We next asked whether CTPI-2 influences insulin sensitivity and glucose tolerance that are impaired in NAFLD/NASH. HFD-fed mice displayed high levels of fasting glucose and circulating insulin, significantly reduced clearance of blood glucose measured with glucose tolerance test (GTT), and were insulin resistant relative to control mice, as revealed by an insulin tolerance test (ITT) (Fig. 3a–d). More importantly, with prolonged exposure to the HFD, vehicle-treated mice became increasingly unable to clear glucose, as documented by the enhanced area under the curve and the spiked levels of glycemia. CTPI-2 reduced the basal glucose levels (Fig. 3a) and induced a complete time-dependent normalization of the GTT and ITT, such that after 11 weeks of treatment the glucose clearance capability of HFD-fed mice was also nearly indistinguishable from that of normally fed mice (Fig. 3c, d).

Thus, CTPI-2 prevents the evolution of steatosis to steatohepatitis and restores glucose homeostasis. In essence, mice treated with CTPI-2 could afford the HFD regimen for prolonged period of times without developing significant liver damage, obesity or disruption of glucose metabolism.

### Slc25a1 is induced by hyperglycemia and is downregulated by CTPI-2 in vivo

The molecular mechanisms that govern Slc25a1 expression in vivo are poorly understood. The analysis of cell extracts derived from the liver and adipose tissues demonstrated that the HFD induced an increase in Slc25a1 protein consistent with previous results (Fig. 3e). Surprisingly, CTPI-2 reduced Slc25a1 expression in the adipose tissue and in the liver. This finding contrasted with earlier studies performed in vitro, where CTPI-2 did not affect the expression levels of the protein but interfered with its mitochondrial citrate transport activity [16]. This result raised the plausible possibility that systemic metabolic changes induced by this agent may be responsible for this effect. We and others have previously shown that in tissue culture cells changes in the insulin and glucose concentration can influence Slc25a1 expression [19, 22–24], thus pointing to the improved glucose homeostasis as one possible culprit for CTPI-2 dependent downregulation of Slc25a1. To test this, we next compared Slc25a1 expression in animals fed either with a normocaloric, high glucose diet (HGD), or a calorie-equivalent low glucose diet that we described previously [25]. The results of these experiments revealed that the expression of Slc25a1 was potently induced in the HGD but was nearly undetectable in LGD-fed animals correlating with the lower levels of fasting glycemia (Fig. 3f, g) and recapitulating the downregulation seen with CTPI-2.

We conclude that in vivo Slc25a1 expression is positively influenced by metabolic cues at least in part mediated by glucose. Together with the finding that CTPI-2 ameliorates glucose metabolism, the data also raise the attractive possibility that CTPI-2 may act as a glucose-restriction mimetic agent.

### CTPI-2 inhibits IL-6 and TNFα production and M1-macrophage polarization

**Fig. 4:**
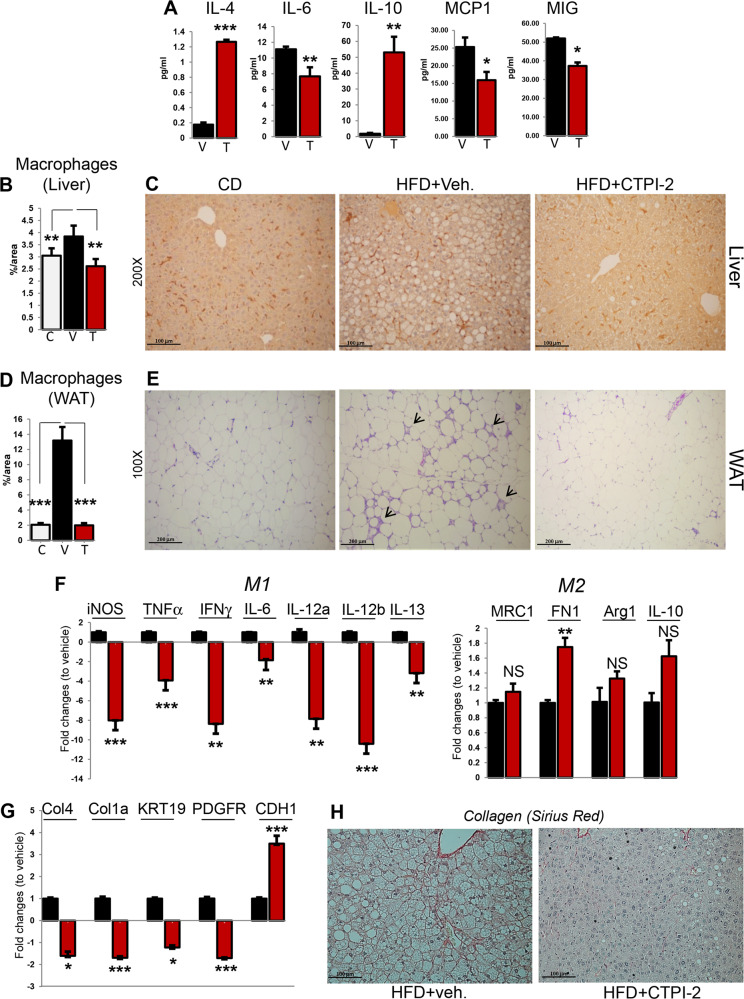
CTPI-2 influences inflammatory pathways. **a** Serum levels of the indicated interleukins and chemoattractant factors from mice fed the HFD and receiving vehicle (V) or CTPI-2 (T). **b** Quantification of liver macrophages in mice fed with control diet (C), or with the HFD and treated with vehicle (V) or CTPI-2 (T). Quantification was performed on 2–3 mice per group and from multiple fields per mouse with the ImageJ program. **c** Representative IHC images of F4/80 staining in the livers of the indicated treatment groups. **d, e** Quantification of macrophages in the WAT (**d**) and representative IHC images of H&E staining in the visceral adipose tissue, with crown structures indicated by arrows (**e**). **f, g** mRNA levels of the indicated genes in the livers of vehicle (black) or CTPI-2 treated mice (red). **h** Representative images of Picro-Sirius Red staining in the liver of the indicated mice. iNOS inducible nitric oxide synthase, TNFα tumor necrosis factor alpha, IFNγ interferon gamma, Interleukins 10–13, MRC1 Macrophage Mannose Receptor 1, FN1 fibronectin 1, Arg1 Arginase 1, Col4 Collagen 4, Col1a Collagen 1a, KRT19 Keratin 19, PDGFR platelet-derived growth factor receptor alpha, CDH1 cadherin-1. *p ≤ 0.05, **p ≤ 0.01, ***p ≤ 0.001.

Liver and adipose tissue recruited macrophages at least in part as a consequence of lipotoxicity, play an important role in tissue damage and the development of fibrotic disease. Macrophages are functionally—though simplistically—divided in M1 and M2. The polarization towards the M1 phenotype contributes to chronic inflammation by promoting the secretion of IL-6, TNFa, and other inflammatory mediators, while M2 macrophages promote the resolution of inflammation and tissue remodeling [26]. Given that CTPI-2 reduced steatosis, we asked whether it also influences inflammation. As shown in Fig. 4a, CTPI-2 lowered the levels of circulating IL-6 while increasing anti-inflammatory IL-4 and IL-10 and also reduced the monocyte chemoattractant protein-1 and monokine-induced by interferon-γ that attract neutrophils and monocytes. Moreover, the HFD increased the amount of Kupffer cells, identified with the F4/80 antibody, and this increase was reversed by CTPI-2 (Fig. 4b, c). The adipose tissue of HFD-fed animals also displayed massive infiltration of macrophages organized in typical crown-like structures and CTPI-2 completely resolved this phenotype (Fig. 4d, e). Consistent with the lower circulating levels of inflammatory molecules, CTPI-2 repressed pro-inflammatory M1 markers, particularly *Tumor Necrosis Factor-a*, *TNFa*, *iNOS*, and *Interleukins*, while either not affecting or slightly increasing M2 markers (*Macrophage Mannose Receptor-1*, *Fibronectin-1*, *Arginase-1*) (Fig. 4f). This was paralleled by a reduction of the expression of the profibrotic genes *Collagen-1/4*, *Keratin-19* and *PDGFR* and by an increase of antifibrotic *Cadherin-1* (Fig. 4g), as well as by a reduction of collagen (Fig. 4h). Thus, CTPI-2 breaks the pro-inflammatory and profibrotic circuits, two important drivers of NASH pathology. Yet, whether CTPI-2 influences the evolution to fibrotic disease needs further evaluation.

### CTPI-2 regulates the citrate pool, the lipogenic and the gluconeogenic pathways

**Fig. 5:**
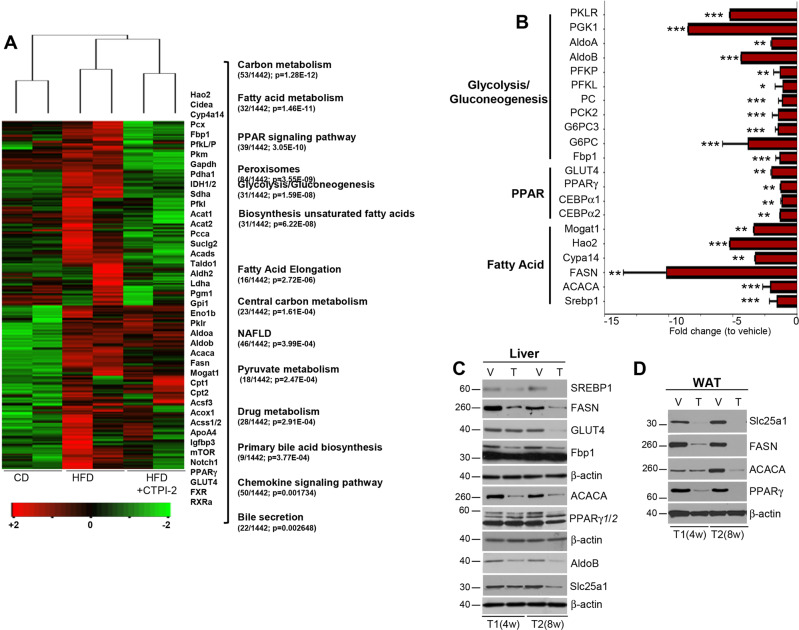
Molecular pathways influenced by CTPI-2. **a** Heat map and partial list of genes identified by RNAseq experiments in the liver of the indicated mice, after 4 weeks of CTPI-2 treatment. Enriched gene sets were identified with KEGG pathway analysis. Numbers in parentheses report the gene ratio and the statistical significance. Relevant genes are shown in the bracket. **b** mRNA levels detected with RT-qPCR of the indicated genes normalized to HFD+vehicle. Most of the reported values are averages of 3 mice per group from independent experiments. The pathways identified with RNAseq and corresponding to the relevant genes are indicated. **c, d** Immuno-blot experiments with the indicated antibodies at the T1 and T2 treatment time points in the liver and WAT. The β-actin levels for each set of blots are shown. *p ≤ 0.05, **p ≤ 0.01, ***p ≤ 0.001.

To identify the molecular mechanisms responsible for the biological effects of CTPI-2, we next performed RNAseq experiments. This approach demonstrated that the HFD induced a large category of transcripts whose expression was attenuated or downregulated by CTPI-2, which essentially reverted the gene expression program induced by the HFD (Fig. 5a). This category included genes involved in fatty acid synthesis *(FASN,* *ACACA*); the *peroxisome proliferator-activated receptors* (*PPAR*) pathway whose members regulate glycolysis, lipid metabolism, and adipogenesis; inflammatory chemokine signal pathways; recently discovered drivers of NASH, particularly *HAO2*, a hydroxyacid oxidase, and monoacylglycerol acyltransferase (*Mogat1*); [27, 28] and a large cluster of genes involved in NAFLD. To validate this analysis, we performed quantitative reverse transcription PCR. This approach concordantly demonstrated a suppression of master lipogenetic genes, including *SREBP1*, *FASN*, and *ACACA* (Fig. 5b). *PPARγ* and its downstream target genes, *CEBPα1*, *CEBPα2*, and *GLUT4*, were also downregulated, while the levels of *PPARα* were unchanged (not shown). This gene expression pattern was matched by changes in the corresponding protein levels in both the liver and adipose tissue (Fig. 5c, d). Furthermore, the RNAseq results showed that CTPI-2 repressed genes/pathways involved in carbon and pyruvate metabolism, in glycolysis, as well as gluconeogenic genes, which are the main contributors to the blood glucose level. These included *Fbp1*, *PC*, *phosphoenolpyruvate carboxykinase* (*PCK1/2*), *glucose 6-phosphatase alpha* (*G6PC*) and *beta* (*G6PC3*), and *Aldolase A/B* (*AldoA/B*) that are limiting for both gluconeogenesis and glycolysis, *PFKL/P*, *Phosphoglycerate Kinase* 1 and *Pyruvate Kinase* (Fig. 5b).

**Fig. 6:**
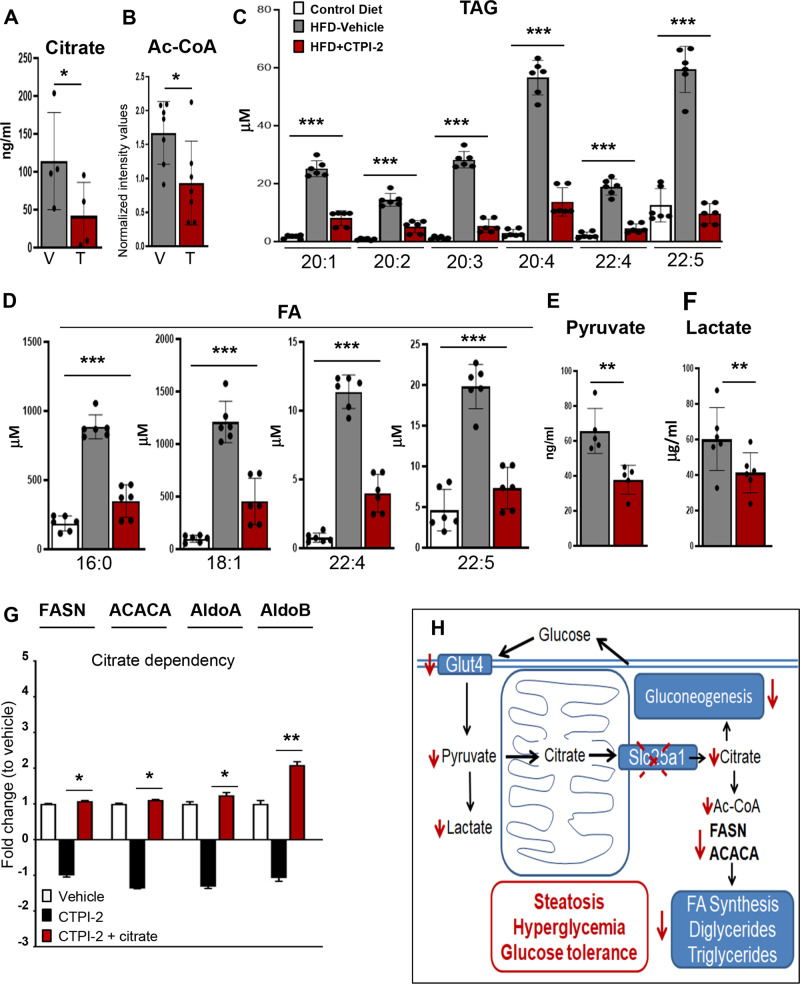
CTPI-2 regulates the citrate concentration in the liver and blunts triglyceride production. **a, b** Citrate and Ac-CoA levels derived from liver extracts of HFD+vehicle (grey) or HFD+CTPI-2 (red). **c, d** Levels of the indicated triacylglycerides (TAG, panel **c**) and total fatty acids (FA, panel **d**) detected with the Lipidyzer platform. *N* = 2–3 mice per group. **e, f** Levels of the indicated metabolites detected with targeted LC-MS analysis. **g** HepG2 cells were treated with CTPI-2 for 5 h, in the presence or absence of 5 mM sodium citrate. The indicated mRNA levels were analyzed with RT-qPCR. **h** Model summarizing our findings (see also text for explanation). Bars indicate standard deviations. *p ≤ 0.05, **p ≤ 0.01, ***p ≤0.001.

Cytoplasmic citrate provides Acetyl-CoA (Ac-CoA) for FA synthesis. Given the role of Slc25a1 in citrate transport, we hypothesized that its inactivation would blunt the availability of citrate and Ac-CoA, thus leading to suppression of the lipogenic program seen with the RNAseq experiments. Indeed, the citrate and Ac-CoA concentrations were reduced in the liver of CTPI-2 treated mice (Fig. 6a, b). Importantly, the levels of gene products that could potentially compensate for citrate deficiency, particularly *Isocitrate Dehydrogenase*s (*IDH1/2*) [29, 30], as well as *Acetyl-CoA synthetases* (*Acss1/2*), that synthesize Ac-CoA from acetate, were reduced by CTPI-2, indicating that Slc25a1 is a key regulator of the hepatic citrate and Ac-CoA pools (Supplementary Fig. S3A–C). An alternative potential contributor to the citrate pool is Indy (I’m-Not-Dead-Yet) or Slc13a5, a cytoplasmic transporter that shares no sequence similarities with Slc25a1 and uptakes citrate from the extracellular space [31]. We found that Slc13a5 mRNA and protein were increased in CTPI-2 treated livers, corresponding with a minimal reduction of the serum concentration of citrate (Supplementary Fig. S3D–F). This result indicates that CTPI-2-treated mice may attempt to compensate for inhibition of Slc25a1 activity by promoting extracellular citrate uptake via this transporter. Moreover, children carrying autosomal recessive mutations of the *Slc25a1* gene develop a severe neurometabolic disorder as a consequence of citrate deficit, hallmarked by the presence of two abnormal products of the TCA cycle, D2-, and L2-hydroxyglutaric acid (2-HG) [9, 10]. However, we were unable to detect elevation(s) of 2-HG either in the serum (not shown) or in the liver of CTPI-2 treated animals (Supplementary Fig. S3G).

Consistent with the reduction of lipogenesis, the levels of the lipid precursors palmitic acid and linoleic acid, of monoglycerides, of diglycerides and of phosphatidylglycerol were significantly diminished (Supplementary Fig. S4) as it was the liver concentration of long-chain triglycerides, the total level of free fatty acids, and particularly those with long-chain bonds containing palmitic-(16:0), stearic-(18:0), arachidonic (20:1), and eicosapentaenoic acid (22:5) (Fig. 6c, d). In addition, pyruvate and lactate, that are products of glycolysis and precursors of gluconeogenesis, were also reduced. In essence, our data are consistent with a deficit very upstream in the lipogenic pathway induced by CTPI-2 and reveal an important role of Slc25a1 in the lipid and glucose metabolism.

To further explore direct effects of CTPI-2 on these genes and on the citrate pool, we next asked (1) how rapidly this agent could regulate their expression in tissue culture cells and, (2) whether such regulation was dependent upon citrate. We found that genes involved in FA synthesis, (*FASN* and *ACACA*), as well as in the glycolytic/gluconeogenesis pathway, (*AldoA* and *AldoB*), were inhibited by a short time treatment with CTPI-2 (5–12 h) and rescued by citrate (Fig. 6g). These experiments not only indicate a direct role for citrate in the regulation of these genes but also highlight the specificity of CTPI-2 for the citrate pathway.

In summary (Fig. 6h), our data have shown that CTPI-2 inhibits glycolysis, PPARγ, and its downstream target the glucose transporter *GLUT4*. The inhibition of glycolysis is manifested with the reduced concentration of pyruvate and lactate. In the liver pyruvate is used for production of citrate in the mitochondria and subsequent export via Slc25a1, promoting lipogenesis through the synthesis of Ac-CoA. Thus, on one side CTPI-2 reduces the availability of citrate and Ac-CoA, reducing precursors for lipogenesis and thereby extinguishing the major steps of de novo FA synthesis, of diacylglyceride and triacylglyceride production. On the other, given that citrate is also an allosteric activator of Fbp1, the reduced concentration of citrate itself or the reduced levels of lactate—which is also a gluconeogenesis precursor—could explain the inhibition of gluconeogenesis. We suggest that the combination of these activities accounts for the beneficial effects of CTPI-2 on steatosis, hyperglycemia, and glucose homeostasis.

### Murine models of *Slc25a1* gene deficiency partially recapitulate CTPI-2 activity

To confirm the role of Slc25a1 in NAFLD/NASH we turned to genetically modified murine models. Mice harboring a germline deletion of the *Slc25a1* allele were initially purchased from the MMRRC. The targeting vector allows for constitutive or conditional deletion of the *Slc25a1* gene through the incorporation of an *IRES*:*lacZ* trapping cassette which is inserted between introns 1 and 5 of the *Slc25a1* gene (*tm1a* allele, Fig. S5A) [32]. Deletion of two copies of the *Slc25a1* gene leads to perinatal lethality and the phenotype of these mice will be described separately. The insertion of the *tm1a* targeting cassette disrupts mRNA translation and *Slc25a1*^*+/−*^ embryos have a 50% reduction of the mRNA and protein levels (Supplementary Fig. S5B–D).

**Fig. 7:**
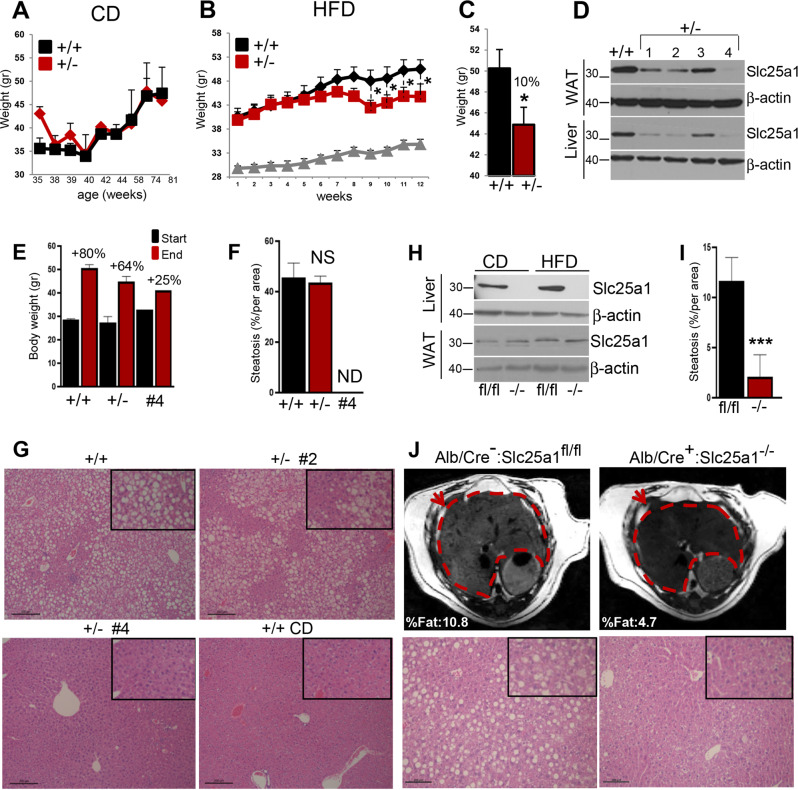
Genetic models of *Slc25a1* gene deficiency partially recapitulate the activity of CTPI-2. **a** Body weight measurements of wild-type or Slc25a1 mice (*n* = 3–6), at different ages (indicated in weeks). **b, c** Body weight of wild-type and Slc25a1 mice in HFD conditions examined over time (**b**), or at the end of experiments (**c**) (*n* = 4). **d**. Immuno-blot experiments of different Slc25a1 mice (indicated from 1 to 4), in the liver and visceral adipose tissue (WAT). **e** Body weight gain (expressed in percentage) of all wild-type and Slc25a1 mice, and of mouse #4. **f, g** Quantification of liver steatosis (**f**), and representative H&E staining (**g**) of the livers of the indicated mice. ND not detected. **h** Immunoblot analysis with the indicated antibodies of the livers and WAT of *Alb/Cre*:*Slc25a1* (fl/fl) and *Alb/Cre:Slc25a1* (−/−) animals in CD and HFD. **i** Quantification of liver steatosis in *Alb/Cre*:*Slc25a1* and *Alb/Cre*:*Slc25a1* animals in HFD conditions. **j** Top panels: magnetic resonance imaging (MRI) of the livers (circled in red and indicated by arrows). Numbers in white indicate the percentage of liver fat calculated with MRI on all slices of the image dataset for each mouse. Bottom panels: representative H&E staining of the livers of *Alb/Cre:Slc25a1* and *Alb/Cre*:*Slc25a1* mice. *p ≤ 0.05, **p ≤ 0.01, ***p ≤ 0.001.

We first analyzed several generations of *Slc25a*1^+/−^ mice for almost 2 years under normal dietetic conditions and found that these mice have body weight comparable with—and live as long as—wild-type mice, demonstrating that the *Slc25a1* gene is not haploinsufficient in the postnatal life (Fig. 7a). However, when fed with the HFD, *Slc25a1*^*+/*^^−^ mice displayed less body weight gain (10%) compared with the wild-type animals (Fig. 7b, c). Furthermore, we observed a significant extent of heterogeneity in the levels of the expression of Slc25a1 protein in the heterozygous mice, as some of these animals showed virtually no protein expression in both the adipose tissue and the liver, compared with others with intermediate loss of expression (e.g., mouse #4, Fig. 7d). Collectively, all heterozygous mice gained less weight under the HFD, but such reduction was significantly more pronounced in mice where Slc25a1 was absent in the WAT (Fig. 7d, e). Conversely, there was no significant effect of *Slc25a1* hemizygosity on steatosis, except for mice with severe depletion of Slc25a1 protein in both the liver and adipose tissue, which were completely protected from steatosis (Fig. 7f, g). These results argue that the activity of Slc25a1 is dose-dependent and rate-limiting in the adipose tissue under conditions of metabolic overload imposed by the HFD,—given that a reduction of dosage herein was sufficient to blunt body weight gain—but not in the liver, where only mice with nearly complete loss of Slc25a1 protein were resistant to steatosis.

Given these results, we next sought to specifically dissect the contribution of liver-expressed Slc25a1. Mice with the targeted insertion of *LacZ* and *Neo* cassettes (*Slc25a1*^*+/−*^) were first crossed with mice expressing the *Flpase* recombinase gene, giving raise to floxed/floxed Slc25a1 alleles (*Slc25a1*^*fl/fl*^, Supplementary Fig. S5E–G) and a generation of these animals was then crossed with *Tg(Alb-cre)21Mgn* (*Alb-Cre*) mice, where the *Cre* gene is under control of the *Albumin* promoter/enhancer elements, generating *Alb/Cre*^*+*^*:Slc25a1*^*−/−*^, as well as control *Alb/Cre*^*−*^*:Slc25a1*^*fl/fl*^ mice. This approach leads to complete loss of the Slc25a1 mRNA and protein in the liver, but not in the adipose tissue, as expected (Fig. 7h). *Alb/Cre*^*+*^*:Slc25a1*^*−/−*^ are born at the expected Mendelian ratio, are viable, and show normal liver histology, body weight and liver fat content (Supplementary Fig. S6A–C). However, when fed the HFD, *Alb/Cre*^*+*^*:Slc25a1*^*−/−*^ mice were largely—although not completely—protected from steatosis assessed with IHC and MRI (Fig. 7i, j and Supplementary Fig. S6D). Furthermore, the analysis of the lipid profile demonstrated a reduction of long-chain triglyceride accumulation (Supplementary Fig. S6E). Similarly to CTPI-2 treatment, *Alb/Cre*^*+*^*:Slc25a1*^*−/−*^ mice showed a clear trend of improvement in the ability to clear blood glucose relative to wild-type mice (Supplementary Fig. S6F).

In conclusion, the phenotypes elicited by CTPI-2 largely overlap with those seen in the genetic models of *Slc25a1* gene deficiency. However, CTPI-2 more effectively prevented steatosis relative to *Alb/Cre*^*+*^*:Slc25a1*^*−/−*^ mice. As discussed later, this difference may depend upon CTPI-2 targeting of Slc25a1 in both the liver and WAT.

## Discussion

The high mortality of NASH is attributed to chronic inflammation and hepatocellular damage that induce a “scar reaction” and the development of fibrosis, which in turn increases the risk of HCC and liver failure [1–4]. Inflammatory signals and macrophages play a key role in NASH evolution. We have shown that CTPI-2 reduces the pro-inflammatory environment systemically and in the liver, inhibiting the M1 pro-inflammatory pathway, reducing macrophage infiltration and dampening the expression of pro-inflammatory and profibrotic genes (Fig. 4). In addition, Slc25a1 promotes the expansion of cancer stem cells and the progenitor stem cell population in the liver is proposed to play an important role in the development of HCC [16, 17, 33, 34]. Collectively, these results grant future studies to elucidate whether CTPI-2 prevents fibrosis and hepatocellular carcinoma.

One important conclusion of our data, viewed collectively, is that the phenotypes elicited by CTPI-2 partially overlap with those seen in the genetic models of *Slc25a1* gene deficiency further corroborating the notion that Slc25a1 is the *bona fide* target of the drug, although we cannot exclude additional targets. Particularly interesting is the observation that heterozygous mice with undetectable levels of Slc25a1 protein in both liver and WAT were almost completely protected from steatosis (Fig. 7). However, the selective deletion of the *Slc25a1* gene in the liver was only partially protective and less efficiently than as seen with CTPI-2, which targets the protein in both tissues. This result re-enforces the importance of the crosstalk between the WAT and liver in the pathogenesis of NASH. The adipose tissue functions as an endocrine system that produces a variety of pro-inflammatory mediators, such as adiponectin, leptin and ghrelin that control appetite, glucose homeostasis, fatty acid oxidation and inflammation [35]. Recent evidence points to a key role for adipose tissue recruited M1 macrophages in the production of TNFα, IL-6, and IL-b1 that we found downregulated by CTPI-2. IL-6 is particularly relevant to NASH as it enhances the Ac-CoA pool, the hepatic glucose production and aggravates steatosis [6, 34–37]. Thus, the efficacy of CTPI-2 most likely stems from its ability to break the dis-metabolic and pro-inflammatory circuits systemically and particularly the crosstalk between the liver and adipose tissues. Testing of this hypothesis will require the generation of additional mouse models harboring the *Slc25a1* gene knockout in multiple tissues.

Various *Slc25a1* gene mutations spanning throughout the coding region occur as either homozygous or compound heterozygous in D2-/L2- hydroxyglutaric (HG) aciduria, a devastating neurometabolic disorder that leads to death often within the first 2 years of life [9, 10, 13]. The enantiomers of 2-hydroxyglutarate (2HG) are proposed to play important roles in regulation of chromatin architecture and cell metabolism [38, 39]. Although the molecular mechanisms by which *Slc25a1* deficiency causes 2-HG aciduria are incompletely clear, at least in *Drosophila*, the accumulation of L-2HG was attributed to the elevation of lactate production arising as a consequence of *Slc25a1* loss of function [40]. By contrast in HFD + CTPI-2 treated mice we could not detect elevated serum or liver concentrations of 2HG and the levels of lactate were decreased both in the liver and serum as a consequence of reduced glycolysis (Supplementary Fig. S3 and Fig. 6), at least in part possibly explaining the lack of 2-HG elevation. Moreover, it is possible that Slc25a1 exerts distinct metabolic functions on the glucose and 2-HG metabolism during development and in the adult life.

In our previous work we have shown that Slc25a1 inhibition depletes the pool of mitochondrial and cytoplasmic citrate, depending upon the cellular context [16]. Cytoplasmic citrate is predominantly derived through export from the mitochondria *via* Slc25a1. A membrane-localized citrate transporter, Slc13a5/INDY, can uptake citrate from the extracellular space contributing to the cytoplasmic concentration of citrate and lipogenesis [31, 41, 42]. However, these two transporters share minimal sequence similarity and, additionally, the -Slc13a5 mediated citrate transport occurs through a different mechanism, being Na-dependent and regulated by the sodium gradient across the membrane [42]. Inhibitors of Slc13a5 have also been identified which show no structure similarity with CTPI-2, but rather exhibit additive effects with Slc25a1 inhibitors [42, 43]. Therefore, it is highly unlikely that CTPI-2 targets Slc13a5 in addition to Slc25a1. On the contrary our data have suggested that Slc13a5 is upregulated at both the mRNA and protein level in the liver of HFD mice treated with CTPI-2 (Supplementary Fig. S3), apparently suggesting that the upregulation of this transporter may provide a mechanism of compensation for Slc25a1 deficiency. An equally important potential mechanism of compensation for citrate production in the absence of functional Slc25a1, consists of production of alpha-ketoglutarate, which is in turn used by IDH1 to produce citrate and to supply Ac-CoA for lipid synthesis [29, 30]. In our RNAseq and immunoblot experiments IDH1 was not induced, but our data do not entirely exclude the possibility that this mechanism of compensation might take place. However, it is also important to note that these previous studies were performed in cells in culture, while ours is the first study that explores the effects of Slc25a1 inhibition in a whole body. Therefore, to what extent these potential mechanisms of compensation take place in a living organism, still needs to be fully investigated.

In summary, our data demonstrate that mice treated with CTPI-2 could afford a high-fat regimen for a prolonged period of time without developing significant liver damage, obesity or disruption of glucose homeostasis. In addition, our results raise the attractive possibility that CTPI-2 may act as a glucose-restriction mimetic agent, potentially expanding its application to pathological conditions that involve disruption of glucose homeostasis. In this study the biological activity of CTPI-2 has proven to be effective and broad, hence framing a strong rationale for further exploitation of pharmacological inhibition of Slc25a1.

### Study approval

All animal studies were approved by the Georgetown University Institutional Animal Care and Use Committee and were conducted accordingly to NIH guidelines.

## Supplementary information

Figure S1

Figure S2

Figure S3

Figure S4

Figure S5

Figure S6

Supplementary Table S1

Supplementary Figure Legends

Supplementary Materials and Methods
