# Supplementary figures and images for: Inhibition of the mitochondrial citrate carrier, Slc25a1, reverts steatosis, glucose intolerance, and inflammation in preclinical models of NAFLD/NASH
Source: Cell Death Differ. 2020 Jan 20;27(7):2143–57. doi: 10.1038/s41418-020-0491-6 (PMC7308387; doi:10.1038/s41418-020-0491-6)

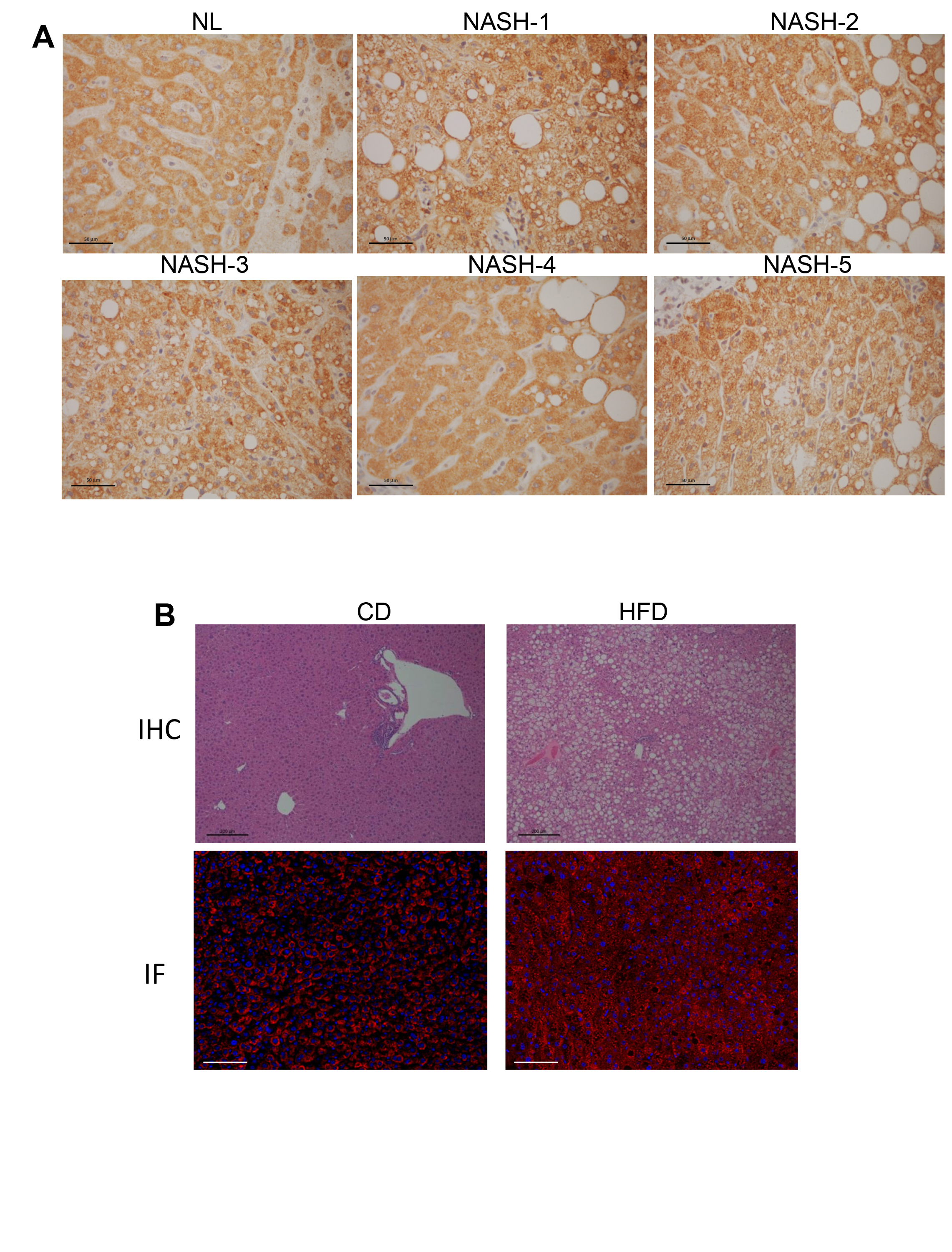

Supplement: Supplementary file 1 — Figure S1 [file 41418_2020_491_MOESM1_ESM.tif]

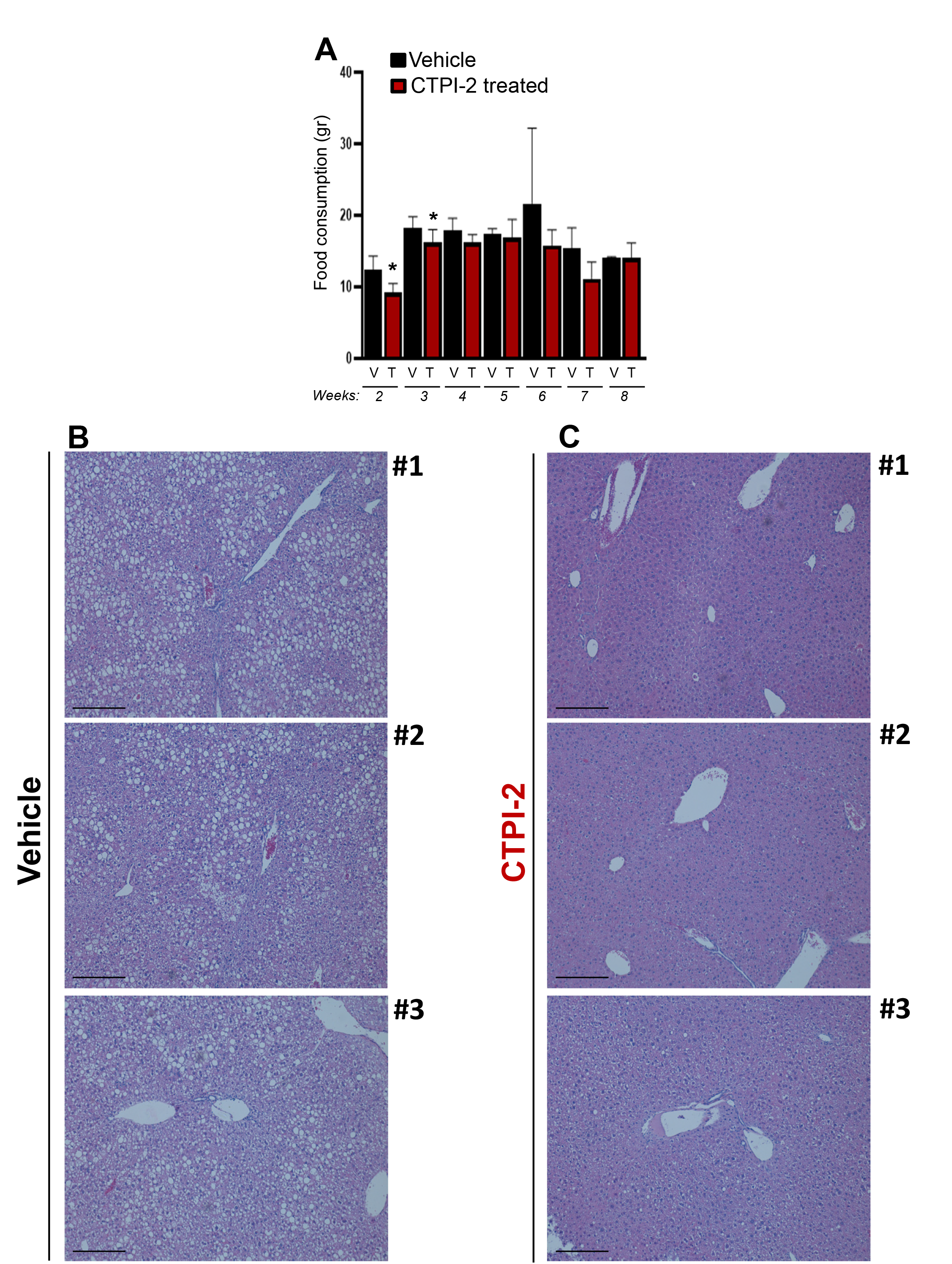

Supplement: Supplementary file 2 — Figure S2 [file 41418_2020_491_MOESM2_ESM.tif]

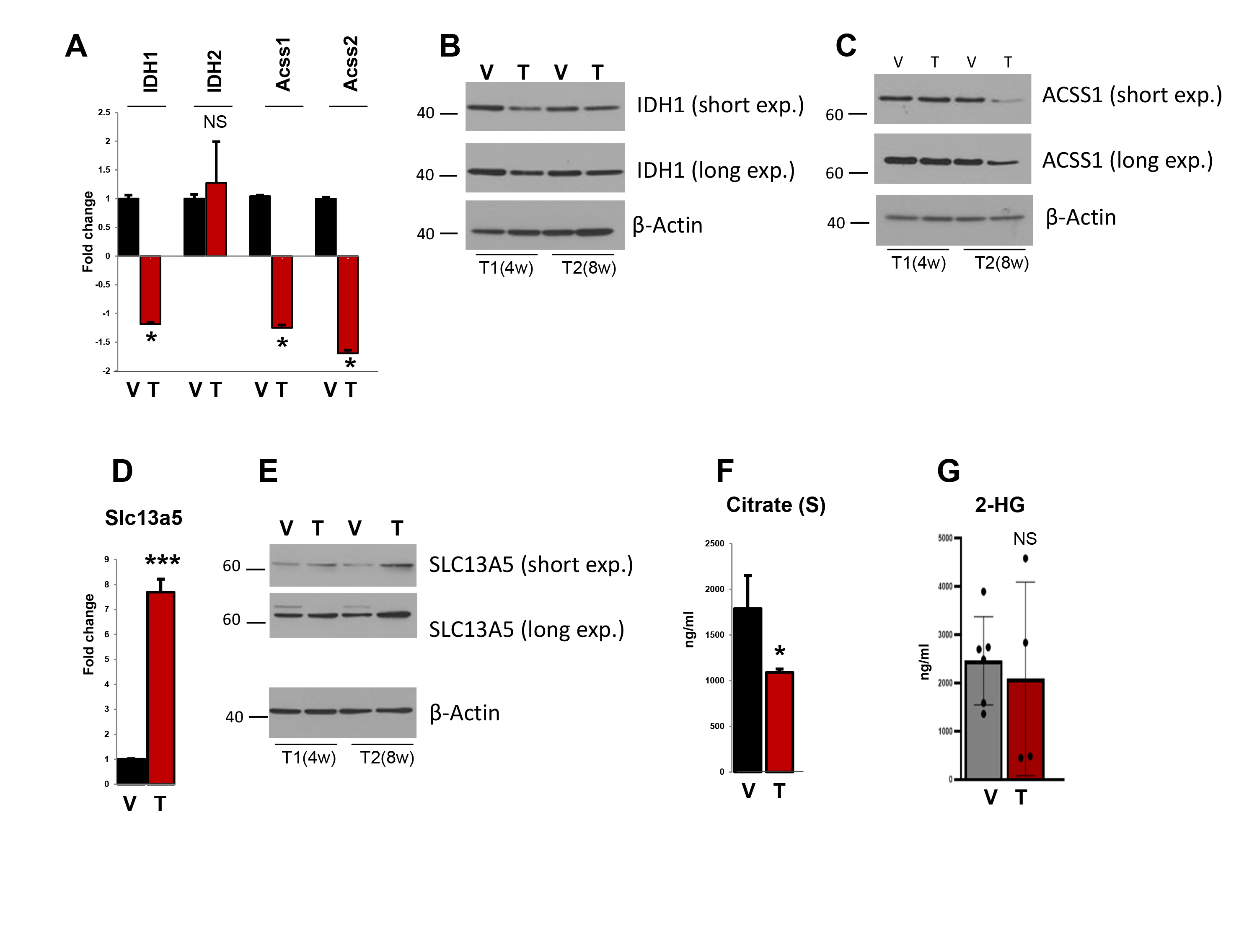

Supplement: Supplementary file 3 — Figure S3 [file 41418_2020_491_MOESM3_ESM.tif]

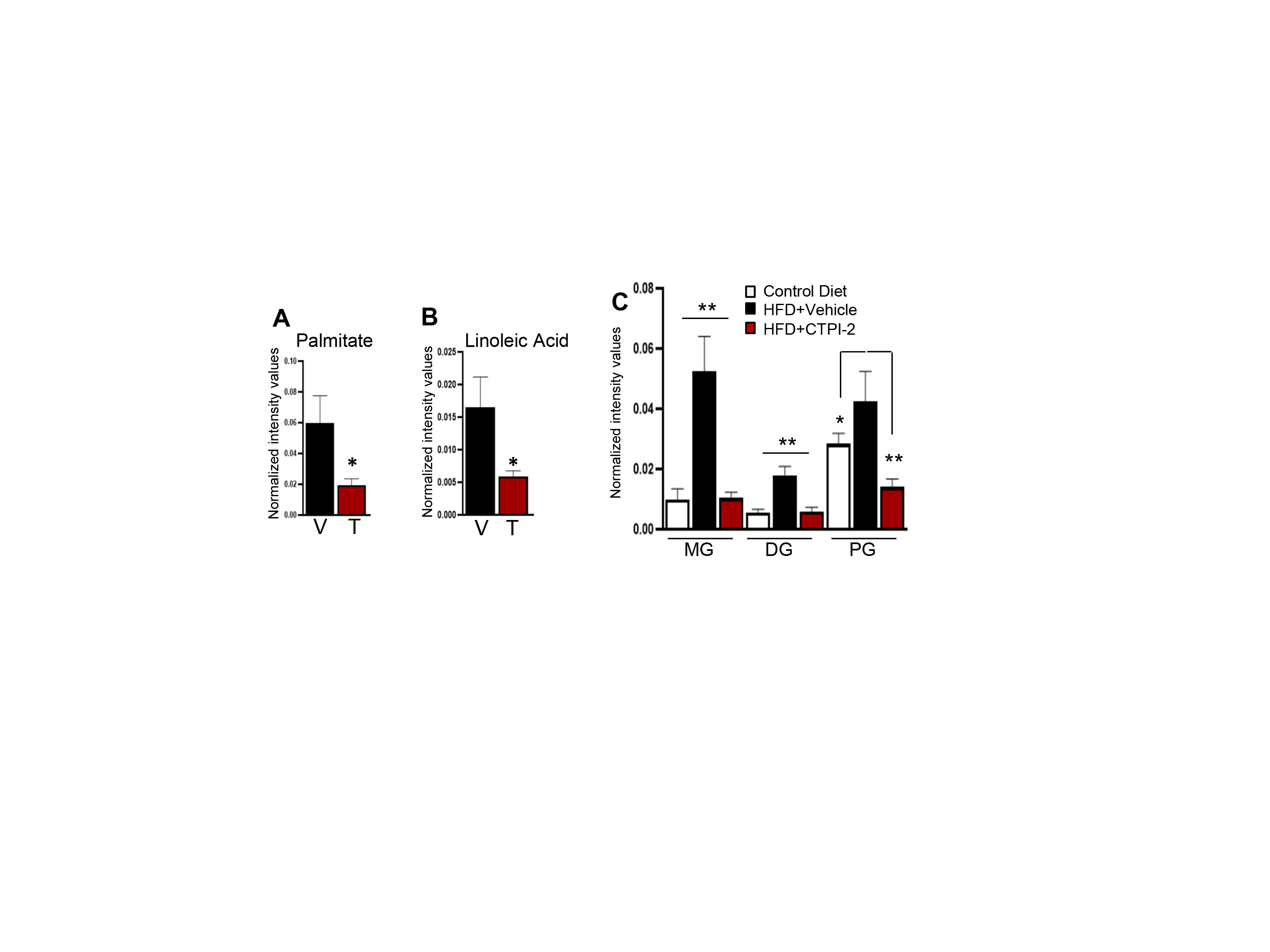

Supplement: Supplementary file 4 — Figure S4 [file 41418_2020_491_MOESM4_ESM.tif]

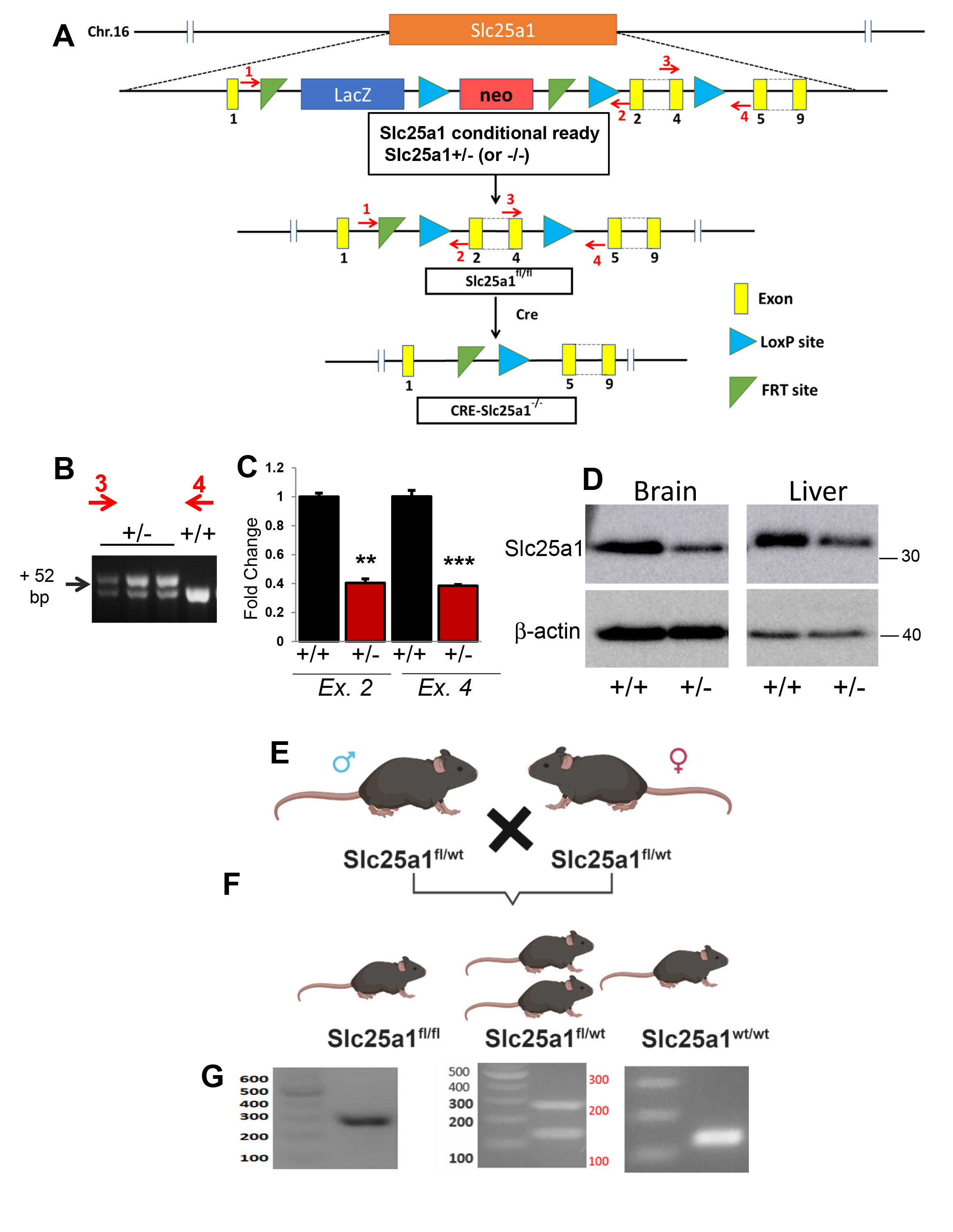

Supplement: Supplementary file 5 — Figure S5 [file 41418_2020_491_MOESM5_ESM.tif]

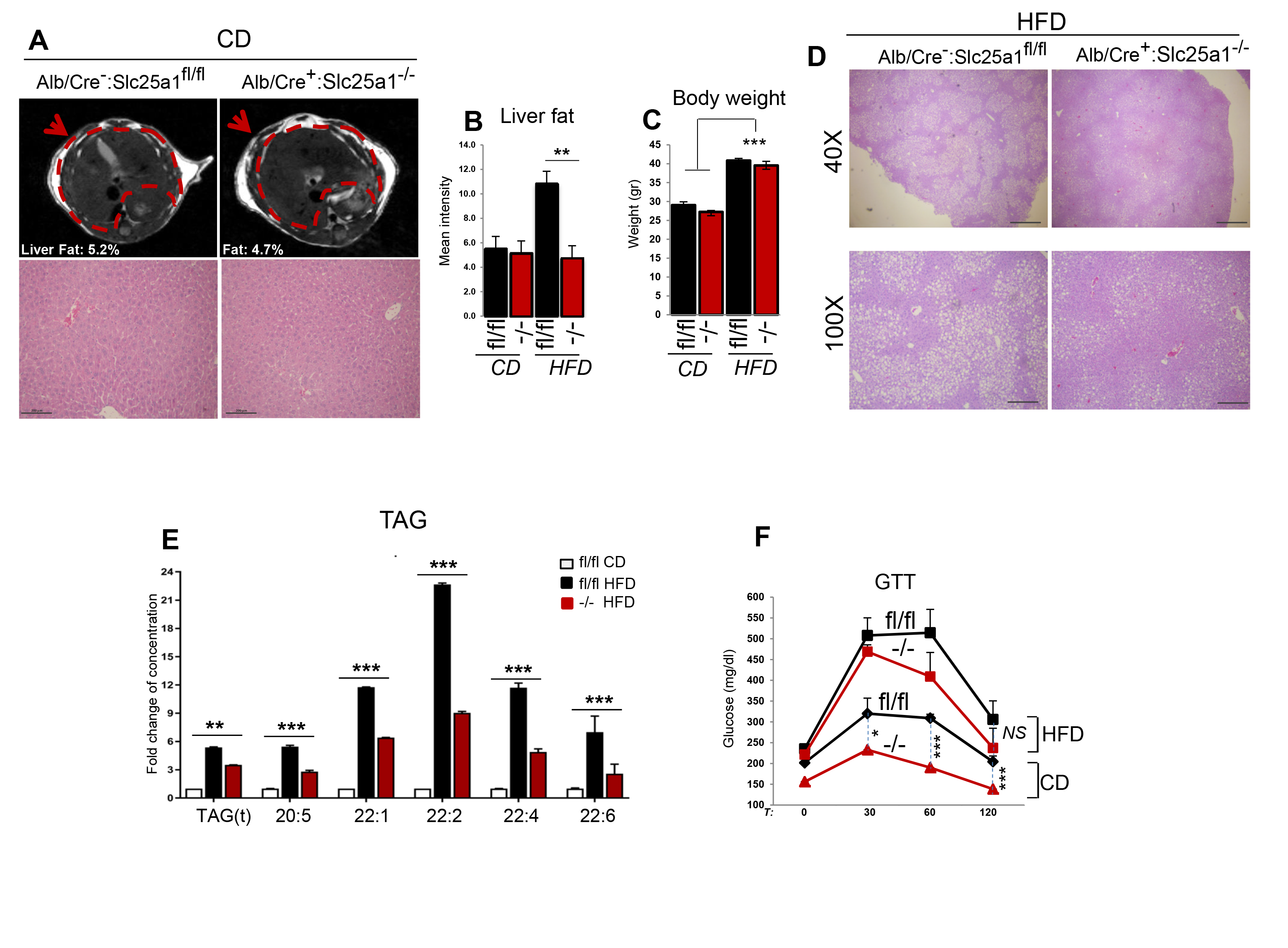

Supplement: Supplementary file 6 — Figure S6 [file 41418_2020_491_MOESM6_ESM.tif]
