# Supplementary material for: Inhibition of the mitochondrial citrate carrier, Slc25a1, reverts steatosis, glucose intolerance, and inflammation in preclinical models of NAFLD/NASH
Source: Cell Death Differ. 2020 Jan 20;27(7):2143–57. doi: 10.1038/s41418-020-0491-6 (PMC7308387; doi:10.1038/s41418-020-0491-6)
Supplement: Supplementary file 7 — Supplementary Table S1 [file 41418_2020_491_MOESM7_ESM.docx]

**Table S1. Murine primers used for the real-time PCR**

| **Genes** | **Forward primer (5’→3’)** | **Reverse primer (5’→3’)** |
| --- | --- | --- |
| *Fbp1* | GGTATGCGCTCTATGGCAGT | TGATGGCAGGGTCAAAGTCC |
| *G6pc* | GGACCTCCTGTGGACTTTGG | TGCTGAGTTCTCCCTTGCAG |
| *G6pc3* | GGGTGCCAGCCTCATGTATT | CGTATCTGGGCATAGCAGGG |
| *Pck2* | TCCAAACTCGCGCTTTTGTG | GTTGAAGGCCTCGTACACCA |
| *Pcx* | TGCCACCAAGATGAGCAGAG | ATGTAGCAACTTCTGGGCCC |
| *Pfkl* | TATGCCCATACGGGTCACAG | CTACCGTGGACCTGGAGAAA |
| *Pfkp* | CAAGCCTTGGTAACCCTCAT | GATGGCAAAGCTATCGGTGT |
| *Aldoa* | GCGATGTCAGACAGCTCCTT | TTAGTCCTTTCGCCTACCCA |
| *Aldob* | CCTTCCCATTGGCAACAA | GAGGACTCTTCCCCTTTGCT |
| *Pgk1* | CAGCCTTGATCCTTTGGTTG | AATGTCGCTTTCCAACAAGC |
| *Pklr* | AATGTTCATCCCTGCCTTGA | GAACACCTCTGCCTTCTGGA |
| *Srebf1* | AGGTGTATTTGCTGGCTTGGT | AGAGATGACTAGGGAACTGTGTGT |
| *Acaca* | GGACCACTGCATGGAATGTTAA | TGAGTGACTGCCGAAACATCTC |
| *Fasn* | GGAGGTTGCTTGGAAGAG | CTGGATGTGATCGAATGCT |
| *Cebpa1* | GCGGGAACGCAACAACATC | GTCACTGGTCAACTCCAGCAC |
| *Cebpa2* | GAACAGCAACGAGTACCGGGTA | GCCATGGCCTTGACCAAGGAG |
| *Pparg* | CCAGAGCATGGTGCCTTCGCT | CAGCAACCATTGGGTCAGCTC |
| *Glut4* | GTGACTGGAACACTGGTCCTA | CCAGCCACGTTGCATTGTAG |
| *Mogat1* | TGGTGCCAGTTTGGTTCCAG | TGCTCTGAGGTCGGGTTCA |
| *Hao2* | GCAGACTTTAAGGCACAAGCA | TGCCAAGTTGTCATTGTAGGTT |
| *Cpt2* | CAGCACAGCATCGTACCCA | TCCCAATGCCGTTCTCAAAAT |
| *Col4a1* | CTGGCACAAAAGGGACGAG | ACGTGGCCGAGAATTTCACC |
| *Col1a1* | GCTCCTCTTAGGGGCCACT | CCACGTCTCACCATTGGGG |
| *Krt19* | CTCCCGAGATTACAACCACTAC | GTTCTGTCTCAAACTTGGTTCTG |
| *Pdgfrb* | ACTACATCTCCAAAGGCAGCACCT | TGTAGAACTGGTCGTTCATGGGCA |
| *Cdh1* | CAGGTCTCCTCATGGCTTTGC | CTTCCGAAAAGAAGGCTGTCC |
| *Nos2 (iNOS)* | GTTCTCAGCCCAACAATACAAGA | GTGGACGGGTCGATGTCAC |
| *Tnf* | CCCTCACACTCAGATCATCTTCT | GCTACGACGTGGGCTACAG |
| *Ifngr1* | ATGAACGCTACACACTGCATC | CCATCCTTTTGCCAGTTCCTC |
| *Il6* | TAGTCCTTCCTACCCCAATTTCC | TTGGTCCTTAGCCACTCCTTC |
| *Il12a* | CTGTGCCTTGGTAGCATCTATG | GCAGAGTCTCGCCATTATGATTC |
| *Il12b* | TGGTTTGCCATCGTTTTGCTG | ACAGGTGAGGTTCACTGTTTCT |
| *Il13* | CCTGGCTCTTGCTTGCCTT | GGTCTTGTGTGATGTTGCTCA |
| *Mrc1* | CTCTGTTCAGCTATTGGACGC | CGGAATTTCTGGGATTCAGCTTC |
| *Fn1* | ATGTGGACCCCTCCTGATAGT | GCCCAGTGATTTCAGCAAAGG |
| *Arg1* | CTCCAAGCCAAAGTCCTTAGAG | AGGAGCTGTCATTAGGGACATC |
| *Il10* | GCTCTTACTGACTGGCATGAG | CGCAGCTCTAGGAGCATGTG |
| *Tbp* | GGGGTCATAGGAGTCATTGG | CATCTCAGCAACCCACACAG |
| *Ppia* | CAGTGCTCAGAGCTCGAAAGT | CACCGTGTTCTTCGACATCA |
| *Actb* | ATGGAGGGGAATACAGCCC | TTCTTTGCAGCTCCTTCGTT |
| *Slc25a1* Exon2 | GAAATCTGCATCACCTTCCCG | GTGGGTTCGCTCGTTCATCT |
| *Slc25a1* Exon4 | TCGAGTTCCTCAGCAACCAC | CCATAGGGCACACGACTACC |
| *Slc25a1^fl/fl^* genoytping (P1+2) | ACTCGGAACCCCAACTCAGA | GACAGCAGACGAGTGGACTTAG |
| *Slc25a1^-/-^* genoytping (P3+4) | tctgttgaaacgacccggag | aggtctccattctccaggct |
| *Alb-Cre* mice genotyping | GAACCTGATGGACATGTTCAGG | AGTGCGTTCGAACGCTAGAGCCTGT |

**Human primers used for the real-time PCR**

| **Genes** | **Forward primer (5’→3’)** | **Reverse primer (5’→3’)** |
| --- | --- | --- |
| *Fasn* | AAGGACCTGTCTAGGTTTGATGC | TGGCTTCATAGGTGACTTCCA |
| *Acaca* | ATGTCTGGCTTGCACCTAGTA | CCCCAAAGCGAGTAACAAATTCT |
| *Aldoa* | TACAGGCACAGTCGCAGAGT | CACTTCCTGGATGCTTGCTG |
| *Aldob* | GATTCATCTGCAGCCAGGAT | AGGAGGACTCTTCTCTCCCAA |
| *SNRPD3* | TCAGCATGTCAGGCAAAATC | GAGGACAACATGAACTGCCA |
| *HSP90AB1* | ATGAGTTGGGCAATTTCTGC | GTCTGGGTATCGGAAAGCAA |

All primers other than specified source are obtained from PrimerBank (https://pga.mgh.harvard.edu/primerbank/) or Primer depot (http://mouseprimerdepot.nci.nih.gov/)
