# Supplementary material for: Inhibition of the mitochondrial citrate carrier, Slc25a1, reverts steatosis, glucose intolerance, and inflammation in preclinical models of NAFLD/NASH
Source: Cell Death Differ. 2020 Jan 20;27(7):2143–57. doi: 10.1038/s41418-020-0491-6 (PMC7308387; doi:10.1038/s41418-020-0491-6)
Supplement: Supplementary file 8 — Supplementary Figure Legends [file 41418_2020_491_MOESM8_ESM.docx]

**Figure S1. Slc25a1 is highly expressed in human and murine NASH livers. A.** Tissue microarrays from human normal liver and NASH patients, expanded from Fig.1B. Data were quantified from 9 NASH samples, 5 of which (NASH-1 to 5) are shown, relative to 3 normal livers. 400X magnification. **B.** IHC (top panels) of mice fed with control- or HFD. Sections from these slides were processed in immuno-fluorescence (IF) with DAPI and the anti-Slc25a1 antibody, and were captured with same settings with a Perkin Elmer Vectra3 Multispectral imaging microscope with a 20X objective (bottom panels). Representative IF images, expanded from Fig.1B are shown.

**Figure S2. Characteristics of CTPI-2 treated mice. A.** Food consumption was measured by weighing the amount of food (in grams) every week over the course of the indicated weeks (n=3-5). To prevent the influence of differences in feeding behavior mice were housed at 1 mouse per cage. **B-C**. Magnification at 100X of HFD-fed mice treated with vehicle (B, left panels) or CTPI-2 (C, right panels) in the prevention study (see also text for explanation). Three mice per group are shown.

**Figure S3. Alternative pathways of citrate and Ac-CoA production. A-C.** mRNA (A) and protein (B,C) of IDH1 and Accs1/2 in the liver of the indicated mice. **D-E**. mRNA (D) and protein (E) levels of Slc13a5. The position of the molecular weight markers is indicated. A short and long exposure of each blots is shown. **F**. Levels (ng/ml) of serum citrate measured with LC/MS. **G**. Levels of 2-hydroxyglutarate (2-HG) measured with LC/MS. Note that this method does not distinguish between the L- or D- form of 2-HG.

**Figure S4. Alterations in the lipid profile induced by CTPI-2. A-B.** Levels of palmitate and linoleic acid in the livers of vehicle (black) or CTPI-2 (red) treated mice, detected with LC-MS. **C**. Monoacyl-glycerol (MG), diacylglycerol (DG) and Phosphoglycerol (PG) levels, in normalized values, obtained from the livers of control diet (white bars), HFD+vehicle (black) and HFD+CTPI-2(red) treated mice, detected with LC-MS. (n=2-3 mice per group).

**Figure S5. Description of the Slc25a1 genetic models. A.** Schematic representation of the Slc25a1 targeting cassette, based on the knock-out first allele (tm1a). In the *Slc25a1* gene on chromosome 16, the cassette is inserted between exon 1 and 5. The position of exon, LoxP and FRT sites is indicated. In red is the position of the primers employed for genotyping (sequences shown in Supplemental Table 1). **B.** Genotyping strategy for the colony of Slc25a1^+/-^ mice. The insertion of the LoxP site between exon 4 and 5 eliminates intron 4 and creates a difference of 52 bp that can be used to discriminate the genotype. The region between exon 4 and 5 was sequenced in the wild-type and mutant animals to confirm the LoxP insertion. **C.** mRNA levels in 19 days whole embryos detected with RT-PCR and using primers spanning within exon 2 and 4 of the murine Slc25a1 mRNA. **D**. Expression levels of Slc25a1 in wild-type and heterozygous mice in the brain and liver of embryos at 19 days post-fertilization. Note that while adult mice do not express Slc25a1 in the brain (Figure 1A), Slc25a1 levels are elevated during embryonic development. **E-F.** Breeding strategy for generating homozygotes *Slc25a1^fl/fl^* mice, as follows: heterozygotes *Slc25a1^fl/wt^* mice were crossed to heterozygotes *Slc25a1^fl/wt^* which resulted in litters of pups containing homozygotes *Slc25a1^fl/fl^*, which were then used for subsequent breeding into *Alb/Cre* mice. **G.** Primers 1 and 2 (panel A, and see sequences in Supplemental table 1) were used to discriminate the genotype, generating an amplicon of 270 bp with the 5’ FRT and loxP site in the *Cre/Slc25a1^fl/fl^*, and 136 bp in the WT allele.

**Figure S6. Phenotype(s) of *Slc25a1* deficient mice. A.** MRI images (top panels) and liver histology (bottom panels) of mice fed with control diet, showing normal liver histology and liver fat content. The red arrows in the MRI images indicate the liver, circled in red. **B.** Quantification of the liver fat content with MRI in the indicated mice. **C.** Body weights of *Alb/Cre^-^:Slc25a1^fl/fl^* or *Alb/Cre^+^:Slc25a1^-/-^* . **D**. Liver of *Alb/Cre^-^:Slc25a1^fl/fl^* or *Alb/Cre^+^:Slc25a1^-/-^* after 12 weeks feeding with HFD. **E**. Total and saturated TAGs levels in animals fed with control diet (white bars), or in *Alb/Cre^-^:Slc25a1^fl/fl^* (black) or *Alb/Cre^+^:Slc25a1^-/-^* (red) animals fed the HFD. **F.** Glucose tolerance test (GTT) in fl/fl *versus* -/- animals in CD or HFD conditions. (n=3). *** p*≤* 0.05*,* ** p*≤* 0.01*,* *** p*≤*0.001. NS: non significant.
