# Supplementary material for: Inhibition of the mitochondrial citrate carrier, Slc25a1, reverts steatosis, glucose intolerance, and inflammation in preclinical models of NAFLD/NASH
Source: Cell Death Differ. 2020 Jan 20;27(7):2143–57. doi: 10.1038/s41418-020-0491-6 (PMC7308387; doi:10.1038/s41418-020-0491-6)
Supplement: Supplementary file 9 — Supplementary Materials and Methods [file 41418_2020_491_MOESM9_ESM.docx]

**Supplementary Materials and Methods**

**Cells, reagents, antibodies, primers**. The CTPI-2 was purchased from Enamine Ltd. The anti-Slc25a1 antibody used in immuno-blot was either from Santa Cruz Biotech, (# [sc-86392](http://www.scbt.com/search/redirect.php?location=datasheet-86392-ctp-t-16-antibody.html&searchPhrase=SLC25A1&datasheet=sc-86392&tableName=&productType=&page=1)) employed at 1:1000 dilution or from Proteintech (15235-1-AP). Additional antibodies were as follows: SREBP-1 (Santa Cruz: sc-13551), ACACA (Cell Signaling: C83B10), FASN (Cell Signaling: C20G5), PPARγ (Cell Signaling:D8I3Y), GLUT4 (Santa Cruz: sc-7938), FBP1 (ABclonal: A5406), ALDOB (Proteintech: 18065-1-AP), β-Actin (Proteintech: 60008-1-Ig) and HSP-70 (Santa Cruz: sc-24).

**Mice and diets.** For the majority of the experiments (except for experiments harboring genetic alterations of the *Slc25a1* gene), C57BL/6J male mice were purchased from Jackson laboratory between 4-6 weeks of age. Mice were acclimated in the GU animal facility for at least one week. At the indicated times, mice were randomized to a standard laboratory chow diet (Labdiet #5053) or the high fat diet (Researchdiets D12492). During the course of the experiments mice were housed at one mouse per cage and food consumption was measured regularly. Animals were maintained in a normal light-cycle and provided with water ad libitum. For the experiments with the high glucose diet mice were randomized to receive a standard laboratory chow diet (C; labdiet #5053), or the low carbohydrates diet (test diet #590N), or the high carbohydrate diet (testdiet #5810).

**CTPI-2 treatment.** CTPI-2 was administered at 50 mg/kg *via* intraperitoneal injection at alternate days. CTPI-2 was diluted either in DMSO (at 0.2% final concentration) using DMSO as vehicle control, or in 0.47% Sodium Bicarbonate (NaHCO_3_) at a final concentration of 14 mM. 0.47% NaHCO_3_ served as vehicle control.

**Glucose and Insulin Tolerance test.** Glucose and insulin tolerance tests (GTT, ITT) were performed by an intraperitoneal injection of glucose (2 g/kg body weight), insulin (0.75 U/kg body weight) in mice following 6 hours fasting (for GTT) or 4 hours fasting (for ITT). Blood was collected from a small incision from the tip of tail before injection (Time 0), then 30, 60 and 120 minutes after injection of glucose or insulin. Blood glucose levels were measured by a blood glucose meter (Accu-Chek^®^ Active, Roche).

**Immuno-blot of murine tissues.** Frozen tissue samples were ground by mortar and pestle in liquid nitrogen and homogenized in RIPA buffer with Roche protease inhibitor cocktail. Protein quantification was done by using Coomassie (Bradford) Protein Assay Kit (Pierce), equal amount of protein lysate was loaded and separated by Novex^™^ 4-20% Tris-Glycine Mini Gel (Invitrogen) then transferred to a PVDF membrane. The membrane was blocked in blocking buffer with 10% horse serum to prevent non-specific binding, followed by incubation with primary antibodies. Appropriate horseradish peroxidase–conjugated secondary antibody (Invitrogen) was applied after incubation with primary antibody, SuperSignal^™^ West Pico PLUS Chemiluminescent Substrate (Thermo Scientific) was used for protein detection. β-Actin or HSP-70 was used as loading control.

**Slc25a1 strains.** The original Slc25a1 tm1a strain was purchased from MMRRC ([C57BL/6N-*Slc25a1^tm1a(EUCOMM)Wtsi^*](https://www.mmrrc.org/catalog/getSDS.php?mmrrc_id=42258), RRID:MMRRC_042258-UCD). The Tg(Alb/cre)21Mgn (Alb-Cre) mice were purchased from Jackson laboratory (#003574). *Slc25a1^+/−^*, *Slc25a1^fl/fl^* and *Alb/Cre* mice were genotyped by conventional PCR with genomic DNA extracted from the One copy of the cassette is present in the mouse genome, which is inserted in Chromosome 16 where the *Slc25a1* gene is located, and targeting confirmation and allele integrity testing were performed by MMRRC. mouse tail biopsies and agarose gel electrophoresis was used for analysis of PCR product. The PCR conditions used were as follows: 95°C for 4 minutes, followed by 40 cycles (95°C for 30 seconds, 65°C for 30 seconds, and 72°C for 50 seconds), 72°C for 2 min then kept in 4°C before use. The PCR primers used for genotyping are indicated in Table S1.

**Histological analysis**. Assessment of steatosis was performed on paraffin embedded H&E stained sections derived from 3-4 animals and on multiple images for animals. The steatosis grade was scored according to the degree of liver involvement with 0: <5%; 1: 5% to 33%; 2: 33% to 50%; and 3: >50%. Alternatively, the percentage of steatosis per area was calculated with ImageJ program.

**Lipidomic analysis.** Frozen tissue samples were weighted (80-100 mg) for analysis and water was added to tissue sample to make total water volume = 1 mL (considering 65% water content in liver tissue). Samples were homogenized and 2 mL of methanol and 900 µL of chloroform were added to the samples. Samples were vortexed and incubated at room temperature for 30 minutes. 1 mL of water and 2 mL of chloroform were added to the extracts and centrifuged at 2000 RPM for 10 minutes. Bottom organic (chloroform) layer was extracted. Extracted chloroform layer was dried under a stream of nitrogen and reconstituted in 250 µL of isopropanol containing 100 ng/ml of splash internal standard mixture. The extracts were transferred into MS vials. Targeted quantitation of triglycerides was performed using multiple reaction monitoring mass spectrometry. The samples were resolved on CORTECS T3 2.7µm, 2.1 x 30 mm column online with a triple quadrupole mass spectrometer (Xevo-TQ-S, Waters Corporation, USA) operating in the multiple reaction monitoring (MRM) mode. Signal intensities from all MRM Q1/Q3 ion pairs for triglycerides were ranked to ensure selection of the most intense precursor and fragment ion pair for MRM-based quantitation. This approach resulted in selection of cone voltages and collision energies that maximized the generation of each fragment ion species. The LC and MRM method used here for the study was developed by Waters cooperation. The metabolite ratios were calculated by normalizing the peak area of endogenous metabolites within tissue samples normalized to the internal standard. Processed data was normalized with respect to tissue weight taken for analysis. The quantitative measurement of lipids with the Lipidyzer platform was performed on the 5500 QTrap with SelexION (Sciex, USA). This method is designed to measure 13 classes of lipid molecules Diacylglycerols (DAG), Chloesterol Esters (CE), Sphingomyelins (SM), Phosphtatidylchloine (PC), Triacylglycerols (TAG), free fatty acids (FFA), ceramides (CE), dihydroceramides (DCER), Hexosylceramide (HCER), Lactosylceramide (LCER), Phosphatidylethanolamine (PE), Lysophatidylcholine (LPC) and Lysophosphatidylethnolamine (LPE) by QTRAP® 5500 LC-MS/MS System (Sciex). The samples were resolved by SCIEX SelexION® technology operating in the multiple reaction monitoring (MRM) mode. Differential mobility spectrometry (DMS) used to separate, hard to resolve ions, to deliver highly-selective, robust and powerful ion separation that significantly enhances the quantitative and qualitative performance. Metabolite dependent parameters e.g. declustering potential, collision voltage, compensation voltage, MRM for 1150 Lipid metabolites are developed by Sciex, complied in ‘Lipidomics Workflow manager software’. The metabolite ratios were calculated by normalizing with respect to labelled internal standard metabolite of each class. The sample queue was randomized and solvent blanks were injected to assess sample carryover. MRM data were processed using ‘Lipidomics Workflow manager software’. The relative quantification values of analytes were determined by calculating the ratio of intensity of transitions of samples normalized to the intensity of the internal standard for 20 infusion cycles.

**Methods for detection of Citrate and TCA cycle intermediates**. Tissue samples were processed using 500 μL of 50% methanol in water containing the internal standard (4-Nitrobenzoic acid, prepared in MeOH at a concentration of 20 µg/mL). The samples were homogenized on ice to ensure tissue lysis and metabolite extraction was then vortexed for 2 minutes. A volume of 10 µL was withdrawn from the homogenized tissue suspension for the protein quantification assays. The samples were vortexed for 2 minutes and left for 2 hours at room temperature to complete the extraction process. The samples were centrifuged (14,000 rpm) at 4°C for 30 minutes and the supernatant was transferred to GC-MS vials and dried under vacuum at room temperature. The BCA kit used for the protein quantification assay was Pierce™ BCA Protein Assay Kit (Cat. # 23225). A volume of 10 µL of each sample of the homogenized tissue suspension in methanol was diluted 10 times with distilled water. A series of standard BSA (Bovine Serum Albumin) was prepared. *Derivatization Method.* A volume of 20 μL of methoxyamine (20 mg/mL) was added to the dried tissue extracts and then heated in an agitator at 60°C for 30 minutes. This was followed by 100 μL of MSTFA. The vials were transferred to an agitator to heat at 60°C for 30 more minutes. Finally, the vials were capped and a volume of 1.5 μL was injected directly to the GC-MS. The samples were allowed to react at room temperature for 20 minutes before being transferred to the GC for injection. Briefly a volume of 1.5 µL of the derivatized solution was injected in (1:10) split mode into an Agilent 7890B GC system (Santa Clara, CA, USA) that was coupled with a Pegasus HT TOF-MS (LECO Corporation, St. Joseph, MI, USA). Separation was achieved on a Rtx-5 w/Integra-Guard capillary column (30 m x 0.25 mm ID, 0.25 μm film thickness; Restek Corporation, Bellefonte, PA, USA), with helium as the carrier gas at a constant flow rate of 0.9 mL/min. The temperature of injection, transfer interface, and ion source were set to 150, 270, and 320°C, respectively. The GC temperature programming was set to 0.2 minutes of isothermal heating at 70°C, followed by 6°C/min oven temperature ramping to 270°C, a 7.0 minute isothermal heating of 270°C, 20°C/min to 320°C, and a 2.0 min. isothermal heating of 320°C. Electron impact ionization (70 eV) at full scan mode (40–600 *m*/*z*) was used, with an acquisition rate of 20 spectra per second in the TOF/MS setting. Peak picking and alignments were performed using ChromaTof 4.7.2 (LECO Corporation). The BCA protein quantitation assay was used to normalize the peak areas. Mass spectra were compared to literature spectra available in the NIST database as well as the Fiehn library of compounds. A pure chemical standard citric acid and oxaloacetic acid was purchased from Sigma-Aldrich, St. Louis, MO (catalog #**251275** and O4126) for validation and calibration.

**RNA isolation, mRNA sequencing and analysis**. Total RNA was isolated from cell pellets using the Direct-zol RNA MiniPrep kit (Zymo Research, USA). The RNA quality and quantity was estimated by UV-VIS spectrophotometry using the NanoDrop ND-1000 spectrophotometer. RNA integrity was assessed using the Agilent RNA 6000 Nano Kit on the Agilent 2100 Bioanalyzer to calculate DV200 (the fraction of RNA molecules >200nt). Final RNA yield and concentration was measured using the Qubit RNA HS Assay Kit, and subsequently normalized to the same concentration across all samples before input. RNA sequencing was performed by the UCLA center for Neurobehavioral Genetics. mRNA libraries were prepared using TruSeq Illumina standard library preparation protocols. mRNA libraries were sequenced on a HiSeq 2500 (Illumina). Adapter trimming and demultiplexing was performed using *cutadapt*. For RNAseq enrichment profiling gene-level quantification was performed using the alignment-free Salmon tool with GRCm38 Mus musculus GENCODE (vM21) annotation. The R package *tximport*was used to summarize to gene level quantification and the R package *DESeq2* was used to perform normalization and differential expression analyses. Hierarchical clustering was used to separate gene expression profiles using the "ward.D" algorithm on euclidean distance matrices. Dendrogram trees were cut and labeled using the *dendextend* R package, and cluster-wise enrichment was calculated using KEGG pathway annotation and the *clusterProfiler* R package.

**Quantitative real-time PCR.** Total RNA was extracted from frozen tissue samples using Trizol reagent (Invitrogen). After genomic DNA treatment by DNase I (Invitrogen), 5µg of total RNA was used in cDNA synthesis with Superscript IV (Invitrogen) and random hexamers according to the manufacturer’s instructions. The real-time PCR reaction on individual cDNAs was performed using the QuantStudio^™^ 12K Flex Real-Time PCR System (Applied Biosystems) with PowerUp^™^ SYBR Green Master Mix (Applied Biosystems). The relative gene expression fold changes calculated using 2^-∆∆CT^ method, were normalized to housekeeping genes *Tbp*, *Ppia* and *Actb*. The forward and reverse primers used are shown in Table S1.

**Immunohistochemistry, immunofluorescence and NASH Tissue Microarrays.** The tissue arrays of human NASH were obtained from XenoTech (1910017). The IHC was performed using standard protocols on formalin- fixed sections. Immunohistochemical staining of mouse liver was performed with Slc25a1 antibody. Five-micron sections from formalin fixed paraffin embedded tissues were de-paraffinized with xylenes and rehydrated through a graded alcohol series. Heat induced epitope retrieval (HIER) was performed by immersing the tissue sections in Target Retrieval Solution, Low pH (DAKO) in the PT Link (DAKO). Briefly, slides were treated with 3% hydrogen peroxide, avidin/biotin blocking, and 10% normal goat serum and exposed to primary antibodies for 1 hour at room temperature. The Slc25a1 antibody (ProteinTech, Cat. 15235-1-AP), was used at 1/150 dilution. Slides were exposed to biotin-conjugated anti-rabbit secondary antibodies (Vector Labs) diluted 1/200 into ImmPress Rat mouse absorbed HRP-conjugated anti-rat secondary antibody (Vector labs, cat. MP-7444). Protein expression was visualized using TSA-488 (Life Technologies, cat. #T20948) and Cy3-SA (Perkin Elmer, cat. #SAT704A001), nuclei visualized with DAPI and the slides mounted in ProLong Anti-fade Gold (Life Technologies P36930). Consecutive sections with the primary antibody omitted were used as negative controls. Stained slides were scanned using the Vectra3 Multi-Spectral Imaging Microscope with Vectra and Phenochart software (Perkin Elmer). The entire slide was scanned, then 10 regions of interest were selected at random throughout the tissue. The scanned images were analyzed in inForm software version 2.4.1.

**Magnetic Resonance Imaging techniques.** *In vivo* magnetic resonance imaging (MRI) of mouse fat depots was performed in the Preclinical Imaging Research Laboratory at the Georgetown-Lombardi Animal Shared Resource and the Center for Translational Imaging in a 7-Tesla horizontal Bruker spectrometer run by Paravision 5.1. Anesthetized (1.5% isoflurane in a gas mixture of 30% oxygen and 70% nitrous oxide) mice were placed in a custom-manufactured (ASI Instruments, Warren, MI) stereotaxic device with built-in temperature and cardio-respiratory monitoring engineered to fit a Bruker mouse volume coil. The sequence used to non-invasively identify adipose tissue is a three-dimensional T1-weighted rapid acquisition with rapid enhancement (RARE) sequences with the following parameters: TR: 250 ms, TE: 34.3 ms, FA: 74.1, Matrix: 156 x 128 x 156.

Quantification of visceral fat depots in the imaging datasets was performed by thresholding and voxel-counting with ImageJ software (NIH). Specifically, we use a maximum intensity projection algorithm of the 3D-reconstructed image with an intensity threshold that shows fat as the brightest signal and disregards signals from other tissues. We calculate the total body fat and separated specific fat deposits in the regions of interest. Liver fat content was determined by placing large ROIs on liver regions and measuring the mean intensity of the fat contrast. Liver ROIs in three slices of each dataset were averaged. Values were normalized to the mean intensity of ROIs placed on background air in each corresponding slice as an internal standard.

**Statistics**. Statistical significance was assessed using both paired or unpaired, two-tailed Student t-test. Significant differences are indicated using the standard Michelin Guide scale (* p< 0.05, significant; ** p< 0.01, highly significant; *** p < 0.001, extremely significant).
